# Phosphorelays Provide Tunable Signal Processing Capabilities for the Cell

**DOI:** 10.1371/journal.pcbi.1003322

**Published:** 2013-11-07

**Authors:** Varun B. Kothamachu, Elisenda Feliu, Carsten Wiuf, Luca Cardelli, Orkun S. Soyer

**Affiliations:** 1 Systems Biology Program, College of Engineering, Computing and Mathematics, University of Exeter, Exeter, United Kingdom; 2 Department of Mathematical Sciences, University of Copenhagen, Universitetsparken 5, Copenhagen, Denmark; 3 Microsoft Research Cambridge, Cambridge, United Kingdom; 4 School of Life Sciences, Gibbet Hill Campus, The University of Warwick, Coventry, United Kingdom; The Centre for Research and Technology, Hellas, Greece

## Abstract

Achieving a complete understanding of cellular signal transduction requires deciphering the relation between structural and biochemical features of a signaling system and the shape of the signal-response relationship it embeds. Using explicit analytical expressions and numerical simulations, we present here this relation for four-layered phosphorelays, which are signaling systems that are ubiquitous in prokaryotes and also found in lower eukaryotes and plants. We derive an analytical expression that relates the shape of the signal-response relationship in a relay to the kinetic rates of forward, reverse phosphorylation and hydrolysis reactions. This reveals a set of mathematical conditions which, when satisfied, dictate the shape of the signal-response relationship. We find that a specific topology also observed in nature can satisfy these conditions in such a way to allow plasticity among hyperbolic and sigmoidal signal-response relationships. Particularly, the shape of the signal-response relationship of this relay topology can be tuned by altering kinetic rates and total protein levels at different parts of the relay. These findings provide an important step towards predicting response dynamics of phosphorelays, and the nature of subsequent physiological responses that they mediate, solely from topological features and few composite measurements; measuring the ratio of reverse and forward phosphorylation rate constants could be sufficient to determine the shape of the signal-response relationship the relay exhibits. Furthermore, they highlight the potential ways in which selective pressures on signal processing could have played a role in the evolution of the observed structural and biochemical characteristic in phosphorelays.

## Author Summary

Two-component phosphorelays constitute the key signaling pathways in all prokaryotes, lower eukaryotes, and plants, where they underline diverse physiological responses such as virulence, cell-cycle progression and sporulation. Despite such prevalence, our understanding of the dynamics and function of these systems remains incomplete. In particular, it is not clear why all phosphorelays studied to date embed a four-layer architecture and how their dynamics could relate to phenotypic variability in the resulting responses. Here, we use analytical approaches and numerical simulations to analyze all possible phosphorelay topologies of length four and embedding reverse phosphorylation. We find that only two topologies can embed both hyperbolic and sigmoidal signal-response relationships, and that one of these can underlie high noise (i.e. phenotypic variability) in population responses. All of the remaining topologies are either non-functional or can embed only a hyperbolic signal-response relationship. Using analytical solutions of relay dynamics, we find that reverse phosphorylation from the third layer, a topological featured commonly observed in nature, is a necessary condition for sigmoidal signal-response relationship.

## Introduction

**Figure 1:**
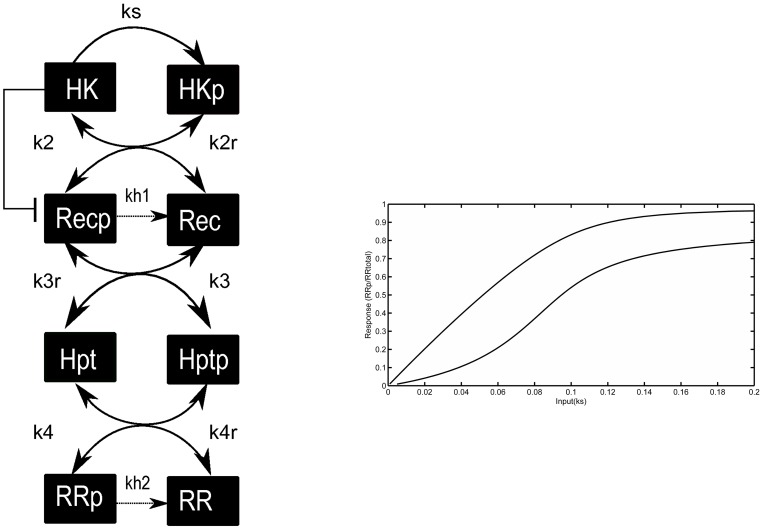
The analysis of phosphorelay topologies and their dynamics. A. Cartoon representation of the general four layered phosphorelay model. Hydrolysis reactions (on aspartate residues found on REC and RR proteins only) and forward and reverse phosphorylation reactions are shown, along with the possibility of the HK being bifunctional. **B.** Hyperbolic and sigmoidal signal-response relationships displayed by a specific topology (topology 30 shown on Figure 2C). The two curves are obtained by choosing specific parameter sets (given in *Supporting Text S5*). The x- and y-axis correspond to the signal and response of the system, which in the model are approximated by the HK auto-phosphorylation rate constant, *k_s_* and by the fraction of phosphorylated RR, respectively.

Biological signaling systems allow cells to produce appropriate physiological responses to external and internal clues. Understanding the signal-response relationships of these systems and how this is shaped by specific biochemical mechanisms is fundamental to predicting and engineering cellular behavior. Among the different signaling systems that cells use, phosphorelays are found in prokaryotes, lower eukaryotes, and plants [1], [2] and are shown to be involved in diverse physiological responses including the regulation of virulence [3], sporulation [4], cell-cycle progression [5], cytokinin signaling [6] and stress responses [7]. Most of the studied phosphorelays to date involve a sequence of four phosphotransfer reactions involving four types of so-called two component proteins [1], [2]. Signal transduction in this four-layered structure starts with the signal-mediated autophosphorylation of the sensor histidine kinase (HK) on a conserved histidine residue. The phosphoryl group is then transferred to an aspartate containing receiver protein (REC), followed by a transfer onto the histidine residue in a phosphotransfer protein (Hpt) and finally to the aspartate residue on a response regulator (RR), which acts as the output of the system (Figure 1). Phosphorylated REC and RR readily undergo hydrolysis reactions due to the inherent instability of phosphorylated aspartate residues [8], [9]. In different systems, these core characteristics are combined with additional features. For example, in some cases there are additional RRs at the end of the relay [5], [10], in other cases HK can function as both a kinase and a phosphatase (bifunctional HK) [5], and some relays are found to be nested within transcriptional feedback loops [11]–[13].

What are the significances, if any, of these structural and biochemical features of phosphorelays? More broadly, what is the functional benefit of having a specific phosphorelay structure for the cell? A widely held view is that phosphorelays have evolved to allow the cell to achieve signal integration using phosphorylation at their different layers [2], [14]. While theoretical studies have shown the potential of relays for signal integration [8], [11], it remains unclear how and why selective pressures on signal integration should lead to the widespread phosphorelay features such as relay length of four and presence of reverse phosphorylation. An alternative possibility is that evolution of phosphorelays has resulted in these specific features due to their effects on signal processing capabilities of the cell. For example, transcriptional feedbacks such as those seen in the *Bacillus subtilis* phosphorelay raise the possibility of achieving bistable dynamics as a functional role of the relay [15], [16]. Recent experimental studies, however, show that this system possesses no bistability and that the embedded feedback loops might have limited effect on its steady state response [12], [13]. One of these studies found high heterogeneity in the phosphorylated RR levels [12], while the other showed experimental evidence for ultrasensitivity in the output of the phosphorelay but suggested that the source of this feature lies downstream of the relay [13]. The former result leads to the proposal that the main functional role of the *B. subtilis* sporulation phosphorelay is to act as a noise generator [12]. It is not clear how these findings and hypotheses should apply to other phosphorelays, and particularly to those that are not nested in transcriptional feedbacks. More generally, functional arguments derived from studies of a specific system are limited in providing a broad understanding of the relation between the observed architectural and biochemical features of phosphorelays and their function.

Achieving such a broader understanding requires mathematical analysis of the signal-response relationship of phosphorelays under a range of alternative biochemical assumptions and parameter ranges. Here, we take this approach and study the role of reverse phosphorylation and hydrolysis reactions in four-layered phosphorelays. Using both numerical simulations and analytical approaches we evaluate the shape of the signal-response relationship in all possible four-layered phosphorelay topologies, arising from distributing hydrolysis and reverse phosphorylation reactions on a base structure. We find that almost half of these topologies are not capable of signal transduction, and further, among those that are, only a few allow more than one type of signal-response relationship. By solving the steady state equations of the system analytically, we find mathematical criteria that relate the total protein levels in different layers and the rate constants of hydrolysis and phosphorylation reactions in a given relay to the shape of the signal-response relationship. In particular, we show that reverse phosphorylation reactions between REC-Hpt and Hpt-RR and hydrolysis at REC and RR enable sigmoidal signal-response relationships in a four-layered phosphorelay that is otherwise confined to displaying only hyperbolic response relationships. Interestingly, these topological features are found in natural four-zlayered phosphorelays. We further show that the ratio of forward and reverse phosphorylation rate constants between REC-Hpt and between Hpt-RR allows tuning the signal-response relationship among the hyperbolic and sigmoidal regimes. In the latter regime, the response of the system to a step signal is faster and noisier compared to the hyperbolic regime, thus enabling subsequent control of the timing and population-level variability of physiological responses. The emerging picture from these analytical and numerical results is that the observed features of four-layered phosphorelays endow them with tunable functionality (i.e. tunable signal-response relationship). These results account for some of the highly conserved topological and biochemical features of phosphorelays and will facilitate experimental determination of signal-response relationship in phosphorelays. In particular, they show that measuring the ratio of reverse and forward phosphorylation rate constants could be sufficient to determine the shape of the signal-response relationship in four-layered phosphorelays.

## Results

To study the role of reverse phosphorylation on the shape of the signal-response relationship, we build a generic mathematical model of a phosphorelay with four layers, which is the observed relay length in all of the commonly studied natural systems studied to date [1]–[7] (see *Methods* and *Supporting Text S1*). The model considers all the forward and reverse phosphotransfer reactions involving HK, REC, Hpt and RR, as well as hydrolysis reactions (Figure 1). While four forward phosphotransfer reactions are necessary to obtain the biologically observed phosphorelay length, the position of reverse phosphotransfer and hydrolysis reactions can be altered to study all theoretically possible relay structures of length four. In total, there are 32 such topologies that embed reverse phosphorylation and hydrolysis reactions in different layers of the relay. These distinct topologies can be derived from a general model (Eq. 1) that includes all of the reactions, by setting specific rate constants to zero (see *Methods* and *Supporting Text S2*).

Using a recently developed recursive technique [17]–[19], we were able to find an analytical description of the steady states of the ordinary differential equations (ODEs) arising from the general model (see *Methods* and *Supporting Text S2*). In particular, an explicit analytical description of the signal-response curve was derived. Analysis of the relations among the concentrations at steady state revealed that 14 of the 32 possible topologies result in non-responsive systems, where the level of phosphorylated RR reaches its maximum for any non-zero signal (see *Supporting Text S2*). Common to all the 14 non-responsive topologies is the absence of hydrolysis reaction on RR (*k_h2_* = 0) and, *additionally*, either there is no hydrolysis reaction on REC (*k_h1_* = 0), or there are no reverse phosphorylation reactions between REC-Hpt (*k_3r_* = 0) or between Hpt-RR (*k_4r_* = 0). In other words, the ability of a four-layered phosphorelay to respond to a range of signals *necessitates* the presence of either hydrolysis from RR, or both hydrolysis from REC and reverse phosphorylation at one of the final two layers.

**Figure 2:**
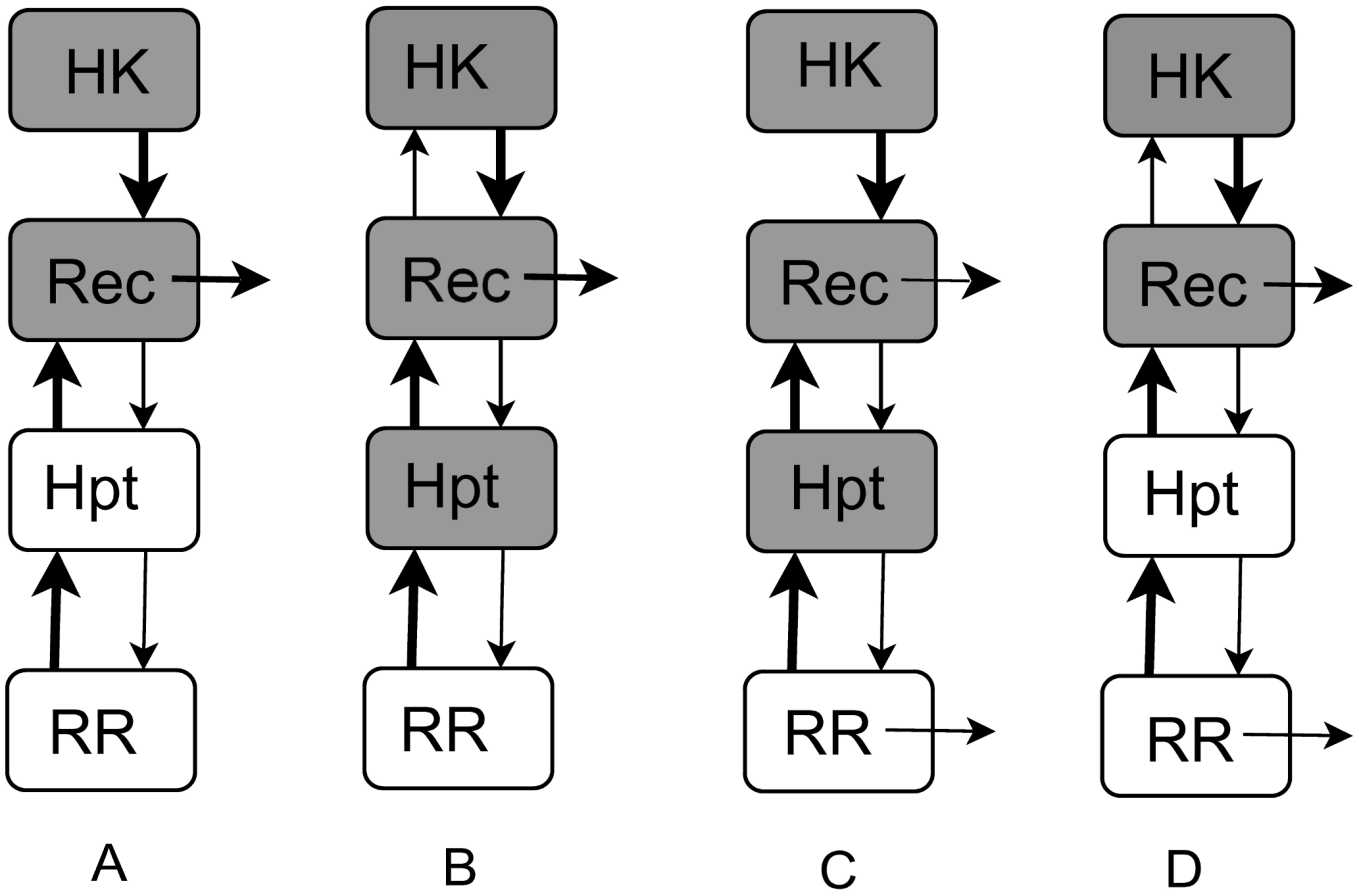
Cartoon representations of the four topologies that allowed sigmoidal signal-response relationships in a significant part (more than 2%) of the sampled parameter space when considering monofunctional HK and different total protein concentrations at different layers (see also **Table 1**, Figure S1 and *Supporting Text S3*). Panels **A**, **B**, **C** and **D** show topologies 14, 16, 30 and 32 respectively, each corresponding to a specific set of reverse phosphotransfer and hydrolysis reactions being present. Reactions are shown as directional arrows, where thickness of the arrow indicates the relative strength of the reaction. In other words, arrows are weighted by the mean reaction rate constant obtained from all sampled parameter sets producing sigmoidality. For each layer and a given topology, a gray (open) backdrop indicates that the mean of total protein concentration at that layer is high (low), based on all sampled parameter sets producing sigmoidal signal-response curves (see *Supporting Text S5* for actual mean parameter values and concentrations).

For the remaining 18 responsive topologies we analyzed the signal-response relationship. For each topology, we have sampled 1000 parameter sets (rate constants and total protein concentrations) from a biologically permissible range, derived the signal-response curve for each parameter set and classified this curve as hyperbolic or sigmoidal (see *Methods*). The hyperbolic case contains linear signal-response relationships with saturation. The sigmoidal case indicates that the signal-response relationship includes an inflection point [20], and could endow the cell with switch-like responses and decision-making [21] via the phosphorelay. A sigmoidal signal-response relationship can also embed ultrasensitivity [20], [22]. The classification of the signal-response curves resulting from parameter sampling revealed that out of the 18 topologies, only 4 allowed sigmoidality in any significant part (more than 2%) of the sampled parameter space and when considering both equal and different total protein concentrations at different layers (see Table 1, Figure 2, and *Supporting Text S3*). Interestingly, common to all these topologies was the presence of reverse phosphorylation between REC-Hpt and between Hpt-RR, which is also observed in the nature. Of the 4 topologies, only two (topologies labeled 14 and 30) resulted in an equal distribution of sigmoidal and hyperbolic responses among the sampled parameter sets suggesting that their signal-response relationship can easily be “tuned” (Table 1).

**Table 1:** The results of the signal-response relationship classification for the 18 responsive topologies.

ID	Reverse Phosphorylation	Hydrolysis	Sigmoid Sets	ID	Reverse Phosphorylation	Hydrolysis	Sigmoid Sets
1	1 0 0	0 1	0.00	16	1 1 1	1 0	15.47
2	0 1 0	0 1	0.00	25	1 0 0	1 1	0.00
3	0 0 1	0 1	0.00	26	0 1 0	1 1	1.53
4	1 0 1	0 1	0.00	27	0 0 1	1 1	0.00
5	1 1 0	0 1	0.00	28	1 0 1	1 1	0.00
6	0 1 1	0 1	0.00	29	1 1 0	1 1	0.33
7	0 0 0	0 1	0.00	30	0 1 1	1 1	44.53
8	1 1 1	0 1	0.00	31	0 0 0	1 1	0.00
14	0 1 1	1 0	50.53	32	1 1 1	1 1	10.47

For each topology, we have sampled 1000 parameter sets (rate constants and total protein concentrations) from a biologically permissible range (*Supporting Text S6*), derived the signal-response curve for each parameter set and classified this curve as hyperbolic or sigmoidal (see *Methods*). The topologies are indicated with a binary identification code that indicates the presence (1) or absence (0) of reverse phosphotransfer reactions along the relay, and the presence (1) or absence (0) of hydrolysis reactions at layers 2 and 4 (see *Supporting Text S1* for all possible topologies). The classification results are given as the fraction of parameter sets resulting in a sigmoidal signal-response curve (the rest being classified as hyperbolic) and assuming a monofunctional HK. Classifications are based on the second derivative of the signal-response curve at zero and from sampling all parameters (with equal total protein concentrations at different layers) (see *Methods*). For additional results using alternative classification and sampling schemes (different total protein concentrations at different layers) or assuming bifunctional HK, see *Supporting Text S3*.

To further understand the effect of reverse phosphorylation in generating sigmoidality, we compared the sampled parameter sets resulting in hyperbolic vs. sigmoidal signal-response relationships. We found that a key difference between the two parameter sets is the ratio between the forward and reverse phosphorylation rate constants, where a mean ratio below one is observed in the case of sigmoidal signal-response relationships (see Table 2, *Supporting Text S4*, and *S5*, and Figures 2 and S1). To analytically confirm if the reverse phosphorylation rate constant being higher than the forward phosphorylation rate constant is a *necessary* condition for achieving sigmoidality in phosphorelays, we computed analytically the second derivative of the signal-response relationship (*Methods* and *Supporting Text S2*). Note that a hyperbolic curve has negative second derivative throughout its domain (in our case, for positive signals), while the second derivative of a sigmoidal curve is initially positive and then it changes sign. Thus, the sign of the second derivative of the signal-response curve at zero can be taken as a test for sigmoidality. This is confirmed also by the agreement of the sigmoidality classifications based on the sign of the second derivative of the signal-response curve at zero and on the entire curve (see *Methods* and *Supporting Text S2* and *S3*). Using the analytical description of the second derivative of the signal-response curve at zero we found three *necessary* analytical conditions for achieving a sigmoidal signal-response relationship: (i) *k_h1_*>0, (ii) *k_2_*>*k_2r_ and* (iii) *k_3r_*>*k_3_ or k_3r_*○+*k_4r_*>*k_3_*○+*k_4_*, where *k_h1_* is the hydrolysis rate for REC and *k_2_* (*k_2r_*), *k_3_* (*k_3r_*) and *k_4_* (*k_4r_*) are the rate constants of forward (reverse) phosphotransfer reactions between HK-REC, REC-Hpt and Hpt-RR respectively. If either (i), (ii) or (iii) are not fulfilled, then the signal-response curve is hyperbolic. As a consequence, *k_h1_* and *k_3r_* are required to be non-zero for sigmoidality to occur. This analytical result is in full agreement with the classification of signal-response curves resulting from parameter sampling. More particularly, the above mathematical conditions explain why only topologies embedding hydrolysis at REC and reverse phosphotransfer between REC-Hpt display sigmoidality, and why only topologies where these reactions are coupled with reverse phosphotransfer between Hpt-RR result in sigmoidality in a larger portion of the parameter space (see Table 1 and Figure 2). We further conclude that the rate constant of the reverse phosphotransfer at HK-REC must be small for sigmoidality to arise (*Supporting Text S2*).

**Table 2:** Mean, minimum and maximum of the ratio of forward to reverse rate constants based on parameter sets resulting in hyperbolic and sigmoidal signal-response relationship.

Parameter	Topology 14		Topology 30	
	Hyperbolic Regime	Sigmoidal Regime	Hyperbolic Regime	Sigmoidal Regime
*<k_3_*/*k_3r_>*	3.519	0.888	5.067	0.960
*min(k_3_*/*k_3r_)*	0.022	0.01	0.016	0.003
*max(k_3_*/*k_3r_)*	234.465	37.073	685.298	27.104
*<k_4_*/*k_4r_>*	3.183	0.927	8.001	0.835
*min(k_4_*/*k_4r_)*	0.004	0.004	0.006	0.004
*max(k_4_*/*k_4r_)*	69.841	47.272	2265.233	13.153

The results shown are for topologies 14 and 30, assuming monofunctional HK, and sampling all parameters (with equal total protein concentrations at different layers). For additional results using alternative classification and sampling schemes (different total protein concentrations at different layers), assuming bifunctional HK, as well as for results from topologies 16 and 32, see *Supporting Text S4*. Mean values of all parameters as found in parameter sets resulting in hyperbolic and sigmoidal signal-response relationships in topologies 14, 16, 30 and 32 are provided as *Supporting Text S5*.

These results can be understood intuitively if we consider the phosphorelay as a set of connected stations, through which phosphoryl groups flow at a rate dictated by the signal strength. Without the presence of reverse phosphorylation and hydrolysis reactions in intermediate layers, phosphoryl groups accumulate at the bottom of the relay at a constant rate, while intermediate layers can remain unphosphorylated until the layers below them are saturated [8], [17]. When hydrolysis from the bottom layer is absent, this saturation effect becomes immediate for the last layer, creating a non-responsive system (as discussed above). Reverse phosphotransfer reactions at layers 3 and 4 generate a back-flow from their respective layers, thereby increasing the signal level required for the saturation of the bottom layer with phosphoryl groups. This buffering effect presents itself in the signal-response curve of a given layer as sigmoidality, where the phosphorylated form in that layer can remain at low levels despite high signal flow from the top of the relay. It can be expected that implementation of subsequent reverse phosphotransfer and hydrolysis reactions from a given layer would increase the buffering effect and result in higher levels of sigmoidality in the signal-response relationship. This intuitive picture is in line with the analytical results described above, which reveal *k_h1_*, *k_2_*, *k_3r_* and *k_4r_* as the key parameters that control the shape of the signal-response relationship (i.e. its sigmoidality) for the last layer (*Supporting Text S2*). It should also be noted that sigmoidality at the last layer of a phosphorelay could still be achieved in relays of shorter or longer length, provided that the general principles outlined above are met through the use of reverse (or cross) phosphotransfer and hydrolysis reactions.

The analyses described so far have several assumptions with regards to modeling phosphorelay dynamics. Firstly, we have assumed bimolecular phosphotransfer reactions without complex formation. This assumption would be satisfied if phosphotransfer reactions, which are distinct from enzyme-driven reactions, happen fast and any complexes formed are short-lived. While there is indication from *in vitro* phosphotransfer reactions that this might be the case (e.g. [23]), we have relaxed the assumption of no complex formation and developed a model that includes complex formation at each layer of the relay. We derived an analytical solution for the second derivative of the signal-response curve at zero for this model (see section 1.5 in the *Supporting Text S2*). By suitable identification of the rate constants of the system without intermediates to the rate constants of the system with intermediates, we obtain that the originally identified condition - that reverse phosphorylation between layers 2–3, and hydrolysis are necessary for sigmoidality - holds when considering complex formation. Secondly, we have assumed constant total protein concentrations in each layer, ignoring the effects of any processes such as expression, degradation and dilution. This assumption would be valid if such processes happen at much slower time scales compared to the signaling dynamics of the relay. Relaxing this assumption and considering production/degradation processes as simple in and out fluxes for un-phosphorylated and phosphorylated proteins respectively, we derived analytically the expression of the second derivative of the signal-response curve at zero (see section 1.6 in the *Supporting Text S2*). Necessary conditions for sigmoidality in this system are *either* that *k_3r_* (reverse phosphotransfer from Hpt to REC) or that *k_4r_* (reverse phosphotransfer from RRp to Hpt) is non-zero. These conditions differ from the necessary conditions for sigmoidality in the simple model. The main differences are that hydrolysis at the second layer (i.e. *k_h1_*>0) is no longer a required condition, and that *k_3r_* can be zero as long as *k_4r_* is not. The first difference arises because the degradation reaction of phosphorylated REC mimics the role of *k_h1_*. Similarly, the second difference is due to the degradation reaction of phosphorylated Hpt and RR in the third and fourth layers. These equalize the roles of the reverse phosphotransfer reactions at the third and fourth layers in controlling the shape of the signal-response curve. Thirdly, we have assumed the absence of auto-dephosphorylation of HK and Hpt. We find that relaxing this assumption and explicitly modeling the auto-dephosphorylation of HK does not alter the conclusions with regards to the necessary conditions for sigmoidality (see section 1.7 in the *Supporting Text S2*). When assuming auto-dephosphorylation of Hpt, we find that the necessary conditions required for sigmoidality are either that the necessary conditions for the simple model hold, or that *k_4r_*>*k_4_* (see section 1.8 in the *Supporting Text S2*). As a consequence, sigmoidality can arise even if *k_h1_* and *k_3r_* are zero, that is, in the absence of hydrolysis at REC and reverse phosphotransfer between Hpt and REC. This can be explained similarly to why degradation reactions alter the necessary conditions for sigmoidality in the simple model.

In the above treatment, we have also assumed a monofunctional HK, while it is known that several HKs can show both phosphorylation and dephosphorylation activity towards their substrate (in this case REC). We find that considering such a bifunctional HK does not alter the overall analytical conclusions regarding the necessity of fast reverse phosphotransfer and presence of hydrolysis reactions for enabling sigmoidality in the system (*Supporting Text S2*). We find that the addition of a bifunctional HK can have significant effects on the distribution of the signal-response relationship classification in specific topologies (*Supporting Text S3*). In particular, topologies with reverse phosphotransfer reactions in all layers exhibit a drop in the number of parameter sets showing sigmoidality when HK is bifunctional, while topologies lacking hydrolysis at REC can exhibit sigmoidality where they could not under the simple model. The latter finding is understandable as the bifunctional HK-mediated dephosphorylation can mimic the effects of hydrolysis at REC (*k_h1_*). We found that varying the rate constant of the HK-mediated dephosphorylation at REC in comparison to changing *k_h1_* has similar but stronger effects on the shape of the signal-response relationship (see Figure S2). The additional dephosphorylation reaction mediated by the bifunctional HK alters the analytical description of the signal-response relationship in such a way that several previously non-responsive topologies become responsive, while the maximal level of phosphorylated RR at steady state remains unaltered (*Supporting Text S2*). However, due to sequestration of phosphorylated REC by the bifunctional HK and subsequent dephosphorylation, a higher amount of signal is required when HK is bifunctional to achieve the same level of response as in the monofunctional case (for a given set of parameters).

**Figure 3:**
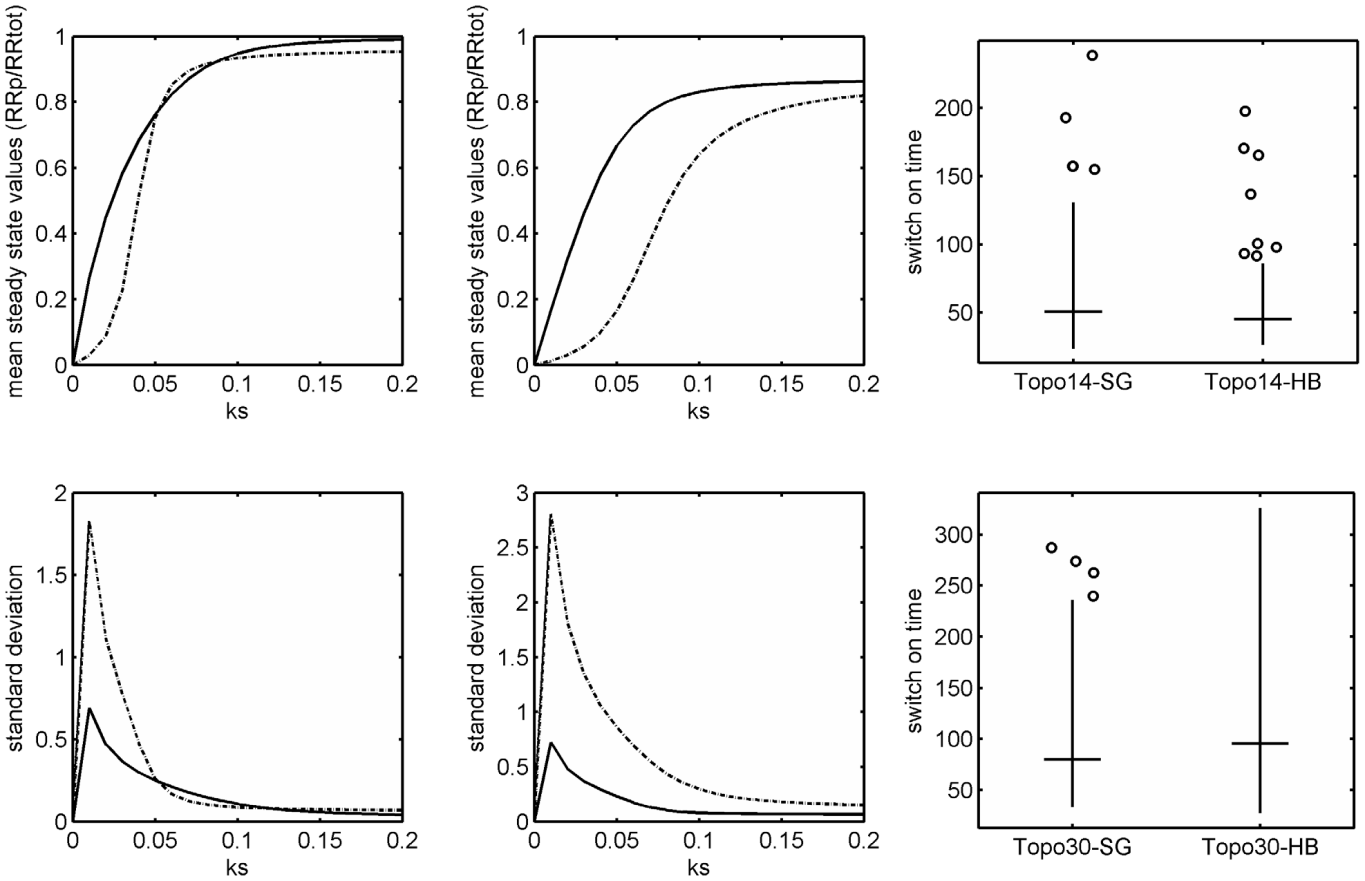
Analysis of noise and response properties of topologies 14 and 30 assuming monofunctional HK. A & B. Signal-response curve for topologies 14 (A) and 30 (B). The x-axis corresponds to the signal input to the system, which in the model is approximated by varying the HK auto-phosphorylation rate constant, *k_s_*. The y-axis corresponds to the simulated mean of the fraction of phosphorylated RR, calculated using PRISM model checker (see *Methods*). The solid and dashed curves show the results obtained from constraining the system parameters in the hyperbolic and sigmoidal regimes respectively. **C & D.** Noise levels in phosphorylated RR for topologies 14 (C) and 30 (D). The x-axis shows the signal input to the system, taken to be the HK auto-phosphorylation rate constant, *k_s_*. The y-axis shows the standard deviation over mean of the fraction of phosphorylated RR at steady state, both calculated using PRISM model checker (see *Methods*). **E & F.** Box plots showing the distribution of response times for topologies 14 (E) and 30 (F) as measured from hyperbolic and sigmoidal regimes. For each topology, the response time is measured for 100 randomly selected parameter sets from the hyperbolic and sigmoidal regimes.

To understand the consequences of hyperbolic vs. sigmoidal signal-response relationship in a phosphorelay, we focused on the two topologies that displayed high levels of tunability between these two response types (topology 14 and 30) and further analyzed the signal-response relationship. As explained above, both of these topologies embed reverse phosphotransfer reactions between REC-Hpt and between Hpt-RR. They differ, however, in the implementation of hydrolysis reactions; topology 30 embeds hydrolysis at the level of both REC and RR, while topology 14 embeds hydrolysis only at the level of REC. For each topology we picked 100 random parameter sets from both hyperbolic and sigmoidal regimes (i.e. parameters resulting in hyperbolic and sigmoidal signal-response relationships), and analyzed the noise properties and response time of the resulting systems (*Methods*). For the noise analysis we considered only stochastic processes *intrinsic* to the relay architecture, e.g. noise arising from binding-unbinding events during phosphotransfer reactions. We find that for both topologies, the phosphorylated RR levels across the full signal range displayed higher levels of noise in the sigmoidal regime compared to the hyperbolic regime (Figure 3). This finding is in line with previous theoretical findings, which showed that the level of noise in a dynamical system is proportional to the level of signal amplification it implements [24].

In contrast to the results from the noise analysis, the results of the response time analysis differed for the two topologies. Response time refers to time required for the phosphorylated RR levels to reach steady state following a step increase (or drop) in signal levels (*Methods*). With the parameters set to result in sigmoidality, topology 30 displayed a smaller response time compared to the case when parameters were set to result in a hyperbolic signal-response relationship (see Figures 3 and S3). For topology 14, however, we did not observe any difference in response times under hyperbolic and sigmoidal regimes. These results can be understood in light of the different hydrolysis reactions present in these two topologies. Having both hydrolysis reactions present, topology 30 displays a lower phosphorylated RR level at saturation and requires a higher signal level to reach this level compared to topology 14 (compare Figure 3A–B). At the same time, however, the presence of both hydrolysis reactions in topology 30 could allow more parameters for tuning the level of sigmoidality and also enhance the effect of the difference between reverse and forward phosphorylation rates from the third layer (see Eq. 2). This could provide the basis for the observed improvement in response time in topology 30, i.e. the ability to achieve sigmoidality via less pronounced reverse phosphorylation rates between Hpt-RR, such that the response at the level of RR is not slowed down.

## Discussion

Phosphorelays are extended two-component signaling systems, which embed additional proteins (or domains) between a HK and RR pair. As such they are the result of evolution exploiting the highly modular nature of two-component proteins [25]. Here, we undertook an analytical and simulation based study to decipher the functional role of the conserved features of these phosphorelays. The findings proved that either the presence of hydrolysis from RR, hydrolysis from REC or reverse phosphorylation between REC-Hpt or Hpt-RR are *necessary* to achieve a responsive four-layered phosphorelay. These structural constraints to achieve a functional signaling system are further refined if the signal-response relationship of such a relay is to be sigmoidal. In particular, we prove that *necessary* conditions for sigmoidality are (i) the presence of hydrolysis at the second layer, (ii) high forward flow of phosphoryl groups at the second layer, and (iii) high reverse flow of phosphoryl groups at the third or both third and final layers. The last conditions indicate that a ratio of forward to reverse phosphorylation rate constants above (below) one strongly favors a sigmoidal (hyperbolic) relationship. The noise characteristics and response times are different in the two regimes resulting in noisier and faster signaling from the phosphorelay when it operates in the sigmoidal regime. We find that bifunctionality of a HK does not alter substantially these conclusions.

These results provide mathematical proof that the way in which hydrolysis and reverse phosphorylation reactions in four-layered phosphorelays is implemented in natural systems endows functionality and could allow tuning of signal-response relationships between a hyperbolic and sigmoidal regime. Together with previous mathematical analyses of phosphorelays, which showed that the maximal level of phosphorylated RR and the signal-to-noise ratio of the response saturate at a relay length of four [8], [17], these findings provide a possible explanation for observed phosphorelay structure. It is plausible that relay length and specific location of reverse phosphorylation and hydrolysis reactions have evolved towards achieving signal processing capability. Evolution could have then exploited specific regimes of rate constants to achieve higher plasticity in the signal-response relationship that phosphorelays could embed. In particular, the phosphorelay structure with reverse phosphorylation between layers 2–3 and 3–4, makes it possible to tune the signal-response relationship of a phosphorelay both through genetic mutations affecting reaction rate constants and through regulatory interactions. The latter could include regulating the total protein concentrations at the different layers of the relay (e.g. via transcriptional regulation or feedback), altering reaction rate constants through binding of auxiliary proteins on relay components, and regulating the bi-functional activity of a HK. Indeed, we find that all these parameters have a significant effect on the shape of the signal-response relationship (Figure S2 and *Supporting Text* S2). There is some empirical evidence that cells might be exploiting such alterations as regulatory points. For example, the kinase and phosphatase activities of certain bifunctional HKs may be regulated through binding of auxiliary ligands [26], [27] or two-component proteins [28]. Experimental studies towards exploring the presence and extent of the other possibilities should be facilitated by the presented results.

These findings are in line with the observations from naturally observed phosphorelays, which are all indicated to display hydrolysis at layers 2 and 4 [1], [2]. Where measured, reverse phosphorylation between layers 2–3 and layers 3–4 are also observed [29], [4], suggesting that topology 30 presented here is common in nature. In the *B. subtilis* sporulation pathway, which is the phosphorelay with most extensive characterization of response dynamics, both reverse phosphorylation reactions are indicated to occur [4], with that between layers 2–3 measured to be strong [30]. Interestingly, this system has been recently shown to display ultrasensitivity [13] and high levels of noise [12]. Our findings indicate that topology 30 can easily rest in a sigmoidal regime and can thus produce both ultrasensitivity and noise. Thus, it is possible that the relay dynamics itself could be the primary source of the experimentally observed dynamics and noise. The *B. subtilis* relay also features transcriptional feedbacks to the second and fourth layers, as well as additional phosphatases, which are indicated to play a role in signal integration [11]. As discussed above, such regulation on the concentrations of relay components could also allow tuning of signal-response relationship between hyperbolic and sigmoidal regimes (see Figure S2).

Considering relay dynamics in light of the findings presented here could help design future experiments to better understand the control of the sporulation decision in *B. subtilis* and the functional roles of other phosphorelays. In particular, the presented findings allow for classification of the signal-response relationship of a phosphorelay from *in vitro* constitution of its parts and measurement of the specific phosphotransfer reaction rates using radiolabelled phosphate groups. Such *in vitro* measurements are commonly employed in the study of bacterial two-component systems [4], [29]–[33], and while they cannot be entirely conclusive about the *in vivo* rates, provide an insight about the parameter regime in which the kinetics of a phosphotransfer reaction resides. Combined with the findings presented here, such measurements would be informative for further experimental designs (e.g. analysis of population level heterogeneity would be interesting to pursue if signaling network dynamics indicates sigmoidal signal-response relationships).

The main conclusions of this study are that phosphorelays can embed hyperbolic or sigmoidal signal-response relationships, and that the latter type is not possible without reverse phosphorylation and a hydrolysis reaction at the second layer. Achieved either via dynamical tuning or through evolution of kinetic rates, the hyperbolic and sigmoidal regimes should allow appropriate physiological responses as needed by the cell. We would expect that sigmoidal dynamics would be favored for responding to signals requiring binary decision making. In contrast, hyperbolic or linear signal-response relationships would be required to produce responses that should track the incoming signals. Classifying a given phosphorelay's behavior into these regimes would be highly valuable, but is currently hampered as measuring the response of a phosphorelay at different signal levels and/or different component concentrations is highly difficult. Further, the signals feeding into phosphorelays are often unknown or not feasible for experimental manipulation. The results presented here offer an alternative, in which the shape of the signal-response relationship of the relay can be predicted from the measurement of forward and reverse phosphorylation rates. These measurements are possible in most cases through *in vitro* phosphotransfer experiments, as discussed above for the *B. subtilis* system, and hence can provide a direct prediction of the *in vivo* signaling dynamics that can be further tested.

Mutations and gene duplications provide the mechanisms by which the structure and dynamics of cellular interaction networks can be changed in evolution. Mathematical and computational approaches such as the ones presented here allow mapping the signal-response relationship of the possible systems that can be generated in this way. This understanding is essential to grasp why evolution might have resulted in the observed features of biological systems and how we might further modulate them. Thus, our findings on phosphorelays should facilitate both understanding the physiology mediated by these systems in a wide range of organisms and (re)engineering these through synthetic biology.

## Methods

### Generic four-layered phosphorelay model

We develop a generic model of a four-layered phosphorelay incorporating all possible combinations of reverse-phosphorylation reactions between layers and hydrolysis reactions (i.e. encompassing all possible topologies in a four-layered relay with reverse phosphorylation and hydrolysis). The hydrolysis reactions are considered possible only on REC and RR, as these proteins are phosphorylated on an aspartate residue (while HK and Hpt are phosphorylated on a histidine residue), which has an inherent instability when phosphorylated [9], [34]. The reactions considered in the model are;
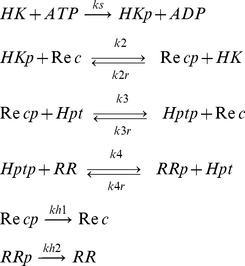
(1)


We initially ignore dimerization of HK and complex formation during phosphotransfer. Further, we consider that dynamics of gene expression and regulation occurs at much slower time scales compared to signaling reactions (e.g. phosphorylation) and hence the protein levels at each layer are assumed to be constant. Assuming mass action kinetics, the dynamics of the concentrations of the species in this reaction system is modeled as a system of ordinary differential equations (*Supporting Text S2*). Steady states are found by setting the derivatives of the concentrations (given by these equations) to zero, together with the constraints imposed by the 4 conserved amounts, one for each layer. By setting certain reaction rate constants to zero, we obtain the steady state equations for the different topologies that embed reverse phosphorylation and hydrolysis at different layers of the phosphorelay. In particular, there are a total of 32 different topologies corresponding to all four-layered phosphorelay structures with reverse phosphorylation and hydrolysis (*Supporting Text S1*).

### Analysis of the signal-response relationship

We analyze the behavior of all possible four-layered phosphorelays using both numerical simulations and an analytical approach. In the latter case, we derived from the steady state equations an analytical expression that relates the level of phosphorylated RR at steady state (i.e. the system output) and the signal level (i.e. system input, taken as the rate of auto-phosphorylation *k_s_*) (*Supporting Text S2*). This expression takes the form *k_s_* = *f* (RR), where the function *f* depends on all the parameters and total protein concentrations of the system except *k_s_*. Specifically, *f* (RR) = RR○+*p_1_* (RR) +*p_2_* (RR)/(*q_1_* (RR) ⋅*q_2_* (RR)), where *p_1_*, *p_2_*, *q_1_*, and *q_2_* are polynomials in RR (*Supporting Text S2*). The signal-response curve is the inverse of *f* and thus to plot the signal-response relationship for a given set of parameters and protein concentrations, we simply plot *f* by plugging in a specific set of parameters and interchange the x- and y-axes (see Figure S4). Further, we use *f* to derive the maximum possible level of phosphorylated RR, *α*, which is computed as the first positive zero of the polynomial *q_2_* (RR) that is derived from *f* (*Supporting Text S2*). For each topology, we have sampled a total of 1000 different parameter sets from a biologically relevant regime (*Supporting Text S6*) and, for each set, constructed the signal-response relationship *f*, i.e. the steady state phosphorylated RR levels corresponding to a given *k_s_* over a range of values of RR between 0 and 0.95○+*α* with increments of *α*/100. We have also derived signal-response curves through numerical simulations, which showed perfect match to analytically derived curves.

### Shape of the signal-response curve

The shape of the signal-response curve carries important information about the response features of a given system [35]. In particular, a curve with a strong sigmoidal shape would indicate switch-like response, whereby signals below a certain threshold do not generate any significant response. While the shape of the signal-response curve has been quantified for specific systems using the ratio of the signals generating 90% and 10% of maximal response [20], measuring the level of “sigmoidality” for any arbitrary signal-response curve is not trivial. Here, we classified each of the signal-response curves resulting from sampling the parameter space in each phosphorelay topology either as sigmoidal or hyperbolic using the second derivative of the inverse of *f* at zero. A sigmoidal curve is a curve whose first derivative is a positive function that initially increases and decreases to zero after an inflection point. Therefore, the second derivative changes sign from positive to negative. A hyperbolic curve is a curve whose first derivative is a positive decreasing function that approaches zero and, consequently, the second derivative is constantly negative. Thus, the sigmoidal or hyperbolic nature of a signal-response curve can be classified using solely the sign of its second derivative at zero. The second derivative of the inverse of *f* at zero can be computed explicitly and checking its sign corresponds to checking the sign of the following expression:
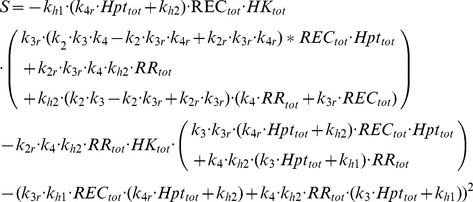
(2)


The derivation of this expression is given in the *Supporting Text S2*. In particular, if *k_h1_* = 0 or *k_2_*<*k_2r_*, or *k_3_*>*k_3r_* and *k_3_k_4_*>*k_3r_k_4r_*, then *S* and hence the second derivative of the signal-response curve at zero are negative and consequently a sigmoidal signal-response relationship is not possible. We have used this criterion to classify the signal-response curves, unless otherwise stated (indicated as “Classification 1” in *Supporting Text S3, S4, S5*). In addition, the second derivative of a given signal-response curve can be calculated numerically and it can be checked if a change of sign occurs along its domain (i.e. over the signal regime it is calculated). We have also used this numerical classification (indicated as “Classification 2” in *Supporting Text S3, S4, S5*), and found that the two approaches give mostly the same results (*Supporting Text S3*). The classified data is then further analyzed to identify parameter regimes leading to hyperbolic vs. sigmoidal signal-response relationships (Table 2 and *Supporting Text S4* and *S5*). We have also considered the signal needed to reach 90% saturation of the maximal response over the signal needed to reach 10% saturation to estimate the signal-response relationships Hill coefficient, *n_H_* = (log 81)/log(input90/input10) [20]. We calculated the *n_H_* for all the generated signal-response curves (i.e. including both hyperbolic and sigmoidal curves) and calculated average values for each topology (*Supporting Text S3*).

### The case with bifunctional HK

In phosphorelays containing a bifunctional HK, un-phosphorylated HK molecules bind to phosphorylated REC and catalyze their dephosphorylation. This reaction extends the system shown in Eq. 1 and results in a new set of ODEs. These new ODEs differ from the original model only in the description of the rate of change for the concentrations of HK and REC, and also incorporate a new species, namely the complex formed by HK and REC (*Supporting Text S2*). We have analyzed the resulting system both analytically (*Supporting Text S2*) and through numerical simulations as described above for the case with monofunctional HK.

### Analysis of noise levels

We utilized the probabilistic model checking approach implemented in PRISM (v4.0.3) [36] to analyze the noise characteristics of topology 14 and 30 under the two parameter regimes corresponding to hyperbolic and sigmoidal signal-response relationships. In brief, this approach generates a continuous time Markov chain model of the system to calculate the probability to be in any of the possible states. While this approach is only feasible for small molecule numbers such that the state space is computationally tractable, it has been shown that the choice of appropriate scaling to ensure computational tractability does not affect the noise characteristics of the system and provides qualitatively similar solutions as obtained from more computationally intensive approaches [37]. To analyze the system with PRISM, we converted the ODE model into elementary reactions with stochastic transition rates. All bimolecular phosphotransfer rates are divided with a scaling factor, *g* = *N_A_*○+V, where *N_A_* is the Avogadro's number and *V* is a volumetric factor with dimensions M^−1^. We set *V* in such a way to ensure 10 molecules for each of the species in the system and thereby limit the number of possible states to a computationally tractable level. For unimolecular reactions, the stochastic transition rates are the same as the mass action rates.

### Analysis of response time

To study the differences in time taken for the relay to respond to a change in input (response time) under the two regimes (sigmoidal vs. hyperbolic), the ODE model arising from topologies 14 and 30 were numerically simulated to steady state under different signal levels. For each topology we picked 100 random parameter sets from each of the sigmoidal and hyperbolic regimes. For each parameter set, we calculated *f* and the maximal response *α* as described above. We have then run a time course analysis where we have simulated a 10% step increase (decrease) starting from a basal signal (i.e. *k_s_*) level of 0.2○+*α*, 0.5○+*α* and 0.8○+*α*. The switch on (off) time is calculated as the time taken for the system to reach a new steady state after the input (*k_s_*) is increased (decreased) (Figures 3 and *S3*). For numerical simulations we used Matlab and its native ODE solvers for stiff systems (ode15s & ode23s). To verify the convergence of the system to steady state, we used the LMFnlsq function, which implements the Levenberg-Marquard-Fletcher method.

## Supporting Information

Figure S1 Cartoon representations of the four topologies shown in Figure 2. Arrows are weighted by the mean reaction rate constant obtained from all sampled parameter sets producing hyperbolic signal-response curves. For each layer and a given topology, a grey (open) backdrop indicates that the mean of total protein concentration at that layer is high (low), based on all sampled parameter sets producing hyperbolic signal-response curves (see Supporting Text S5 for actual mean parameter values and concentrations for these four topologies). Panels **A**, **B**, **C** and **D** show topologies 14, 16, 30 and 32 respectively, each corresponding to a specific set of reverse phosphotransfer and hydrolysis reactions being present. Reactions are shown as directional arrows, where thickness of the arrow indicates the relative strength of the reaction.
(TIF)

Figure S2 Effects of key model parameters on signal-response curves. Panels (**A** & **B**) show the effects of varying *k_5_* (dashed line) and *k_h1_* (dotted line) on the signal-response curves in both the sigmoidal and hyperbolic regimes. Panels (**C**, **D**, **E** and **F**) show that the shape of the signal-response curve can be tuned from one regime to another by varying total protein levels at different layers of the relay. The x-axis is the signal to the system (the HK auto-phosphorylation rate constant, *k_s_*), while the y-axis corresponds to the concentration of phosphorylated RR. Each line represents a system with varying total protein levels. Parameters used for the control curve are as follows (given in the order; *k_2_*, *k_3_*, *k_4_*, *k_2r_*, *k_3r_*, *k_4r_*, *k_h1_*, *k_h2_*, *k_5_*, *k_5r_*, *k_6_*, HK_tot_, REC_tot_, Hpt_tot_, RR_tot_): **A**: (9343, 30201, 35826, 0, 7192, 99251, 0.0302, 0.00234, 1000, 0.012, 3.5, 1.2748e-04, 1.5755e-04, 1.3634e-04, 1.2516e-04); **B**: (186860, 90605, 35827, 0, 35963, 49626, 0.0302, 0.0023, 1000, 0.012, 3.5, 1.2748e-04, 1.5755e-04, 1.363e-04, 1.251e-04); **C**: (5, 0.1, 0.01, 0, 10, 0.10, 10, 0.001,-,-,-, 5, 1, 1, 10); **D**: (500, 0.1, 0.01, 0, 1, 0.10, 0.1, 0.001,-,-,-, 1, 0.005, 100000, 100); **E**: (5000, 0.1, 1, 0, 1, 0, 10, 100, 0, -,-,-,1, 10, 2000, 1); **F**: (5000, 0.1, 0.01, 0, 1, 0.001, 10, 1,-,-,-, 1, 0.1, 1, 30).
(TIF)

Figure S3 Analysis of the response dynamics in topologies 14 and 30. Box plots show the distribution of response off times for topologies 14 (A) and 30 (B) as measured from hyperbolic and sigmoidal regimes. Response off time is defined as the time taken for the system to reach a new steady state after the input (*k_s_*) is decreased by 10% (see Methods). For each topology, the response off time is measured for 100 randomly selected parameter sets from the hyperbolic and sigmoidal regimes.
(TIF)

Figure S4 Plot of *f* and the signal-response curve (inverse of *f*) for select parameter sets. **A.** Plot of *f* for values of RR between 0 and the maximal response α. **B.** The value of *k_s_* corresponding to a given value of RR. **C.** Plot of the signal-response curve. The parameter sets used to create these figures and the expression of *f* are given in the *Supporting Material S2*.
(PDF)

Text S1 List of all possible topologies in a four-layered phosphorelay. The topologies are indicated with a binary identification code that indicates the presence (1) or absence (0) of reverse phosphotransfer reactions along the layer, and the presence (1) or absence (0) of hydrolysis reactions at layers 2 and 4.
(XLS)

Text S2 Derivation of the analytical results and proofs.
(PDF)

Text S3 The results of the signal-response relationship classification for the 18 responsive topologies using different classification and sampling schemes (equal or different total protein concentrations at different layers), or assuming mono- or bi-functional HK.
(XLS)

Text S4 Mean of the ratio of forward to reverse rate constants based on samples resulting in hyperbolic and sigmoidal signal-response relationship in topologies 14, 16, 30 and 32. All results are based on different classification and sampling schemes (equal or different total protein concentrations at different layers), and assuming mono- or bi-functional HK are shown.
(XLSX)

Text S5 Mean values of the parameters resulting in hyperbolic and sigmoidal signal-response curves in topologies 14, 16, 30 and 32. The results are based on different classification and sampling schemes (equal or different total protein concentrations at different layers), and assuming mono- or bi-functional HK.
(XLSX)

Text S6 The “biologically relevant” parameter regime for the parameters of the model and references to the experimental studies, from which this information is compiled.
(XLSX)
