# Supplementary figures and images for: Phosphorelays Provide Tunable Signal Processing Capabilities for the Cell
Source: PLoS Comput Biol. 2013 Nov 7;9(11):e1003322. doi: 10.1371/journal.pcbi.1003322 (PMC3820541; doi:10.1371/journal.pcbi.1003322)

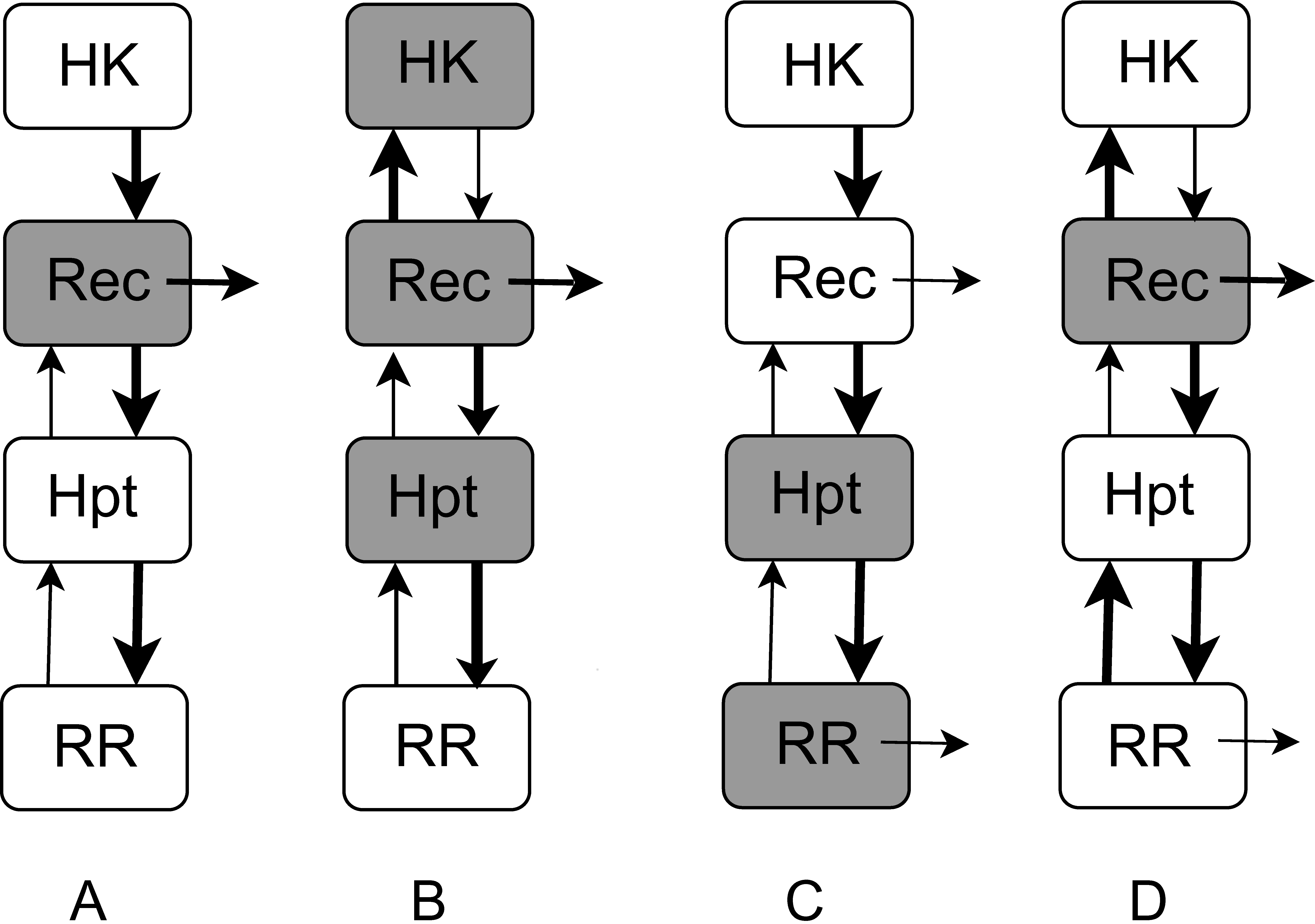

Supplement: Figure S1 — Cartoon representations of the four topologies shown in Figure 2. Arrows are weighted by the mean reaction rate constant obtained from all sampled parameter sets producing hyperbolic signal-response curves. For each layer and a given topology, a grey (open) backdrop indicates that the mean of total protein concentration at that layer is high (low), based on all sampled parameter sets producing hyperbolic signal-response curves (see Supporting Text S5 for actual mean parameter values and concentrations for these four topologies). Panels A, B, C and D show topologies 14, 16, 30 and 32 respectively, each corresponding to a specific set of reverse phosphotransfer and hydrolysis reactions being present. Reactions are shown as directional arrows, where thickness of the arrow indicates the relative strength of the reaction. (TIF) [file pcbi.1003322.s001.tif]

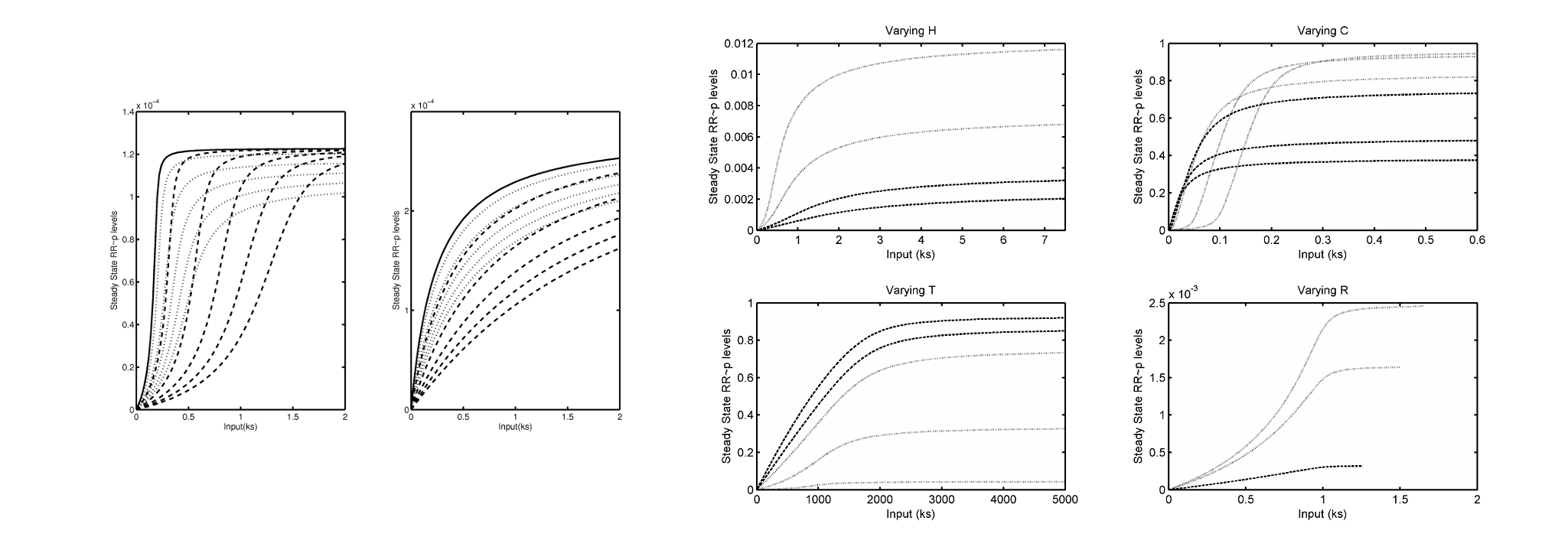

Supplement: Figure S2 — Effects of key model parameters on signal-response curves. Panels (A & B) show the effects of varying k5 (dashed line) and kh1 (dotted line) on the signal-response curves in both the sigmoidal and hyperbolic regimes. Panels (C, D, E and F) show that the shape of the signal-response curve can be tuned from one regime to another by varying total protein levels at different layers of the relay. The x-axis is the signal to the system (the HK auto-phosphorylation rate constant, ks), while the y-axis corresponds to the concentration of phosphorylated RR. Each line represents a system with varying total protein levels. Parameters used for the control curve are as follows (given in the order; k2, k3, k4, k2r, k3r, k4r, kh1, kh2, k5, k5r, k6, HKtot, RECtot, Hpttot, RRtot): A: (9343, 30201, 35826, 0, 7192, 99251, 0.0302, 0.00234, 1000, 0.012, 3.5, 1.2748e-04, 1.5755e-04, 1.3634e-04, 1.2516e-04); B: (186860, 90605, 35827, 0, 35963, 49626, 0.0302, 0.0023, 1000, 0.012, 3.5, 1.2748e-04, 1.5755e-04, 1.363e-04, 1.251e-04); C: (5, 0.1, 0.01, 0, 10, 0.10, 10, 0.001,-,-,-, 5, 1, 1, 10); D: (500, 0.1, 0.01, 0, 1, 0.10, 0.1, 0.001,-,-,-, 1, 0.005, 100000, 100); E: (5000, 0.1, 1, 0, 1, 0, 10, 100, 0, -,-,-,1, 10, 2000, 1); F: (5000, 0.1, 0.01, 0, 1, 0.001, 10, 1,-,-,-, 1, 0.1, 1, 30). (TIF) [file pcbi.1003322.s002.tif]

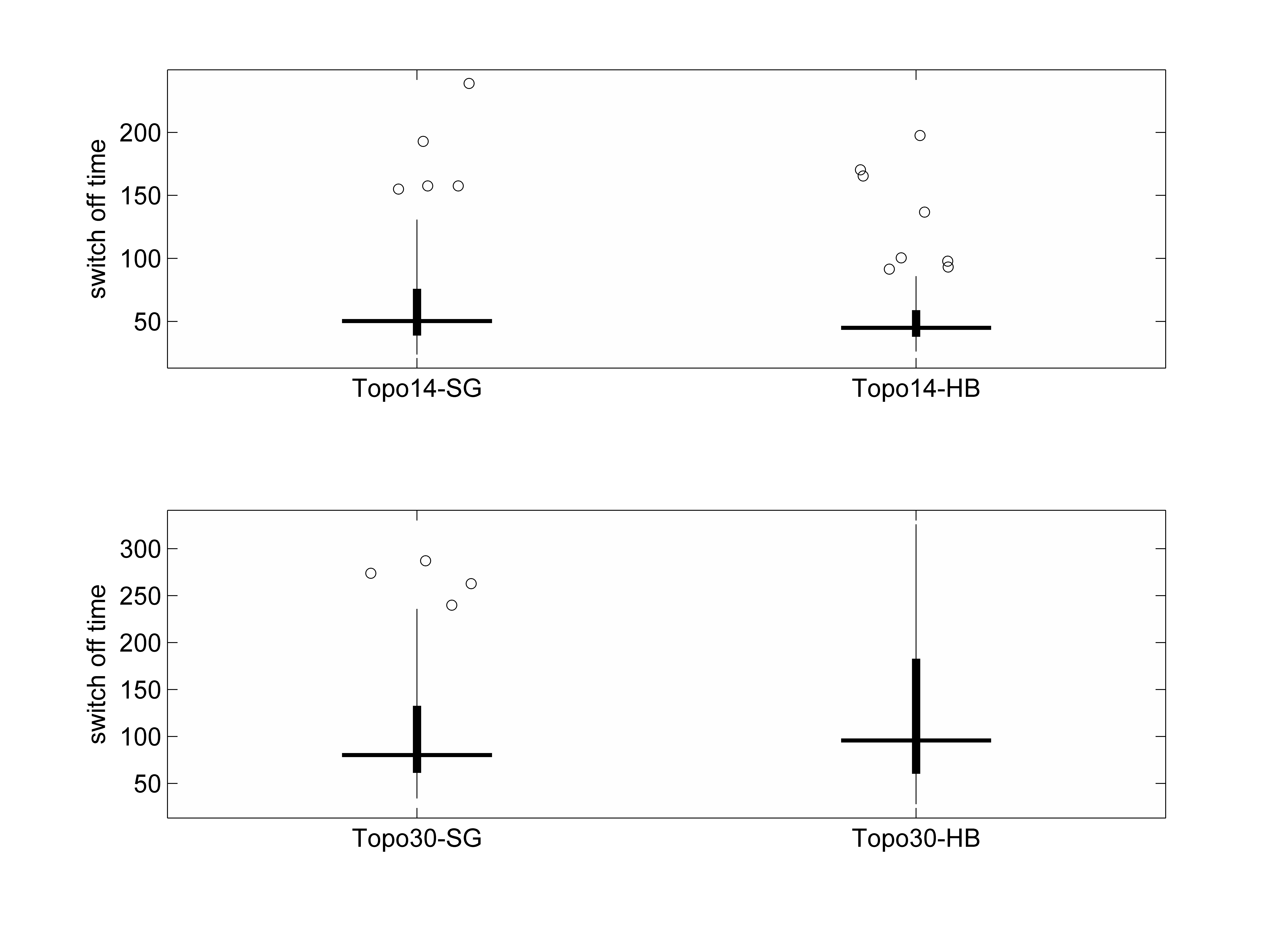

Supplement: Figure S3 — Analysis of the response dynamics in topologies 14 and 30. Box plots show the distribution of response off times for topologies 14 (A) and 30 (B) as measured from hyperbolic and sigmoidal regimes. Response off time is defined as the time taken for the system to reach a new steady state after the input (ks) is decreased by 10% (see Methods). For each topology, the response off time is measured for 100 randomly selected parameter sets from the hyperbolic and sigmoidal regimes. (TIF) [file pcbi.1003322.s003.tif]

A

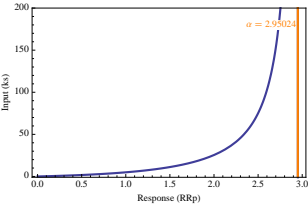

B

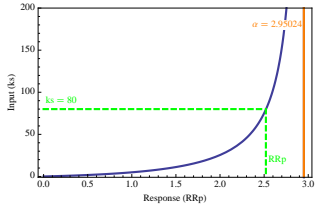

C

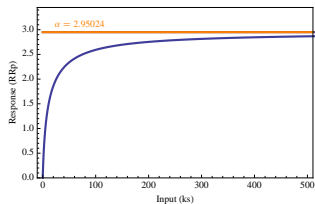

Supplement: Figure S4 — Plot of f and the signal-response curve (inverse of f) for select parameter sets. A. Plot of f for values of RR between 0 and the maximal response α. B. The value of ks corresponding to a given value of RR. C. Plot of the signal-response curve. The parameter sets used to create these figures and the expression of f are given in the Supporting Material S2. (PDF) [file pcbi.1003322.s004.pdf]
