# Supplementary material for: Phosphorelays Provide Tunable Signal Processing Capabilities for the Cell
Source: PLoS Comput Biol. 2013 Nov 7;9(11):e1003322. doi: 10.1371/journal.pcbi.1003322 (PMC3820541; doi:10.1371/journal.pcbi.1003322)
Supplement: Text S2 — Derivation of the analytical results and proofs. (PDF) [file pcbi.1003322.s006.pdf]

Phosphorelays provide tunable signal processing capabilities for the cell

Supplementary Text

Varun B Kothamachu, Elisenda Feliu, Carsten Wiuf, Luca Cardelli, Orkun S Soyer

18/08/2013

This document provides the proofs and explanations of the analytical results described in the main text. The results are summarized in the first two sections and the claims are proven in the two appendices.

Contents

1 The kinase HK is monofunctional **2**
- 1.1 Reactions, equations and steady states . . . 2
- 1.2 Steady-state relations and signal-response curve . . . 4
- 1.3 Hyperbolic and sigmoidal signal-response curves . . . 6
- 1.4 Examples . . . 8
- 1.5 Model with intermediates . . . 8
- 1.6 Model with production and degradation . . . 12
- 1.7 Model with auto-dephosphorylation at HK . . . 13
- 1.8 Model with auto-dephosphorylation at Hpt . . . 14

2 The kinase HK is bifunctional **14**
- 2.1 Reactions, equations and steady states . . . 14
- 2.2 Steady-state relations and signal-response curve . . . 16
- 2.3 Hyperbolic and sigmoidal signal-response curves . . . 19

A Proof of the claims: monofunctional case **20**
- A.1 Constant signal-response curves and zero solutions . . . 20
- A.2 Steady-state relations . . . 21
- A.3 Signal-response curve . . . 22
- A.4 Hyperbolic shape when phosphorelay rates are large . . . 22

B Proof of the claims: bifunctional case **23**
- B.1 Zero concentrations . . . 23
- B.2 Steady-state relations . . . 24
- B.3 Signal-response curve . . . 26

1 The kinase HK is monofunctional

1.1 Reactions, equations and steady states

The model consists of four proteins: a histidine kinase HK, a receiver protein REC, a His-containing phosphotransfer protein Hpt, and a response regulator RR. Each of these proteins can be either phosphorylated (in which case we write Xp, where X is one of the four proteins HK, REC, Hpt, and RR) or unphosphorylated (in which case we write X).

Reactions. The minimal set of reactions that the system has, consists of the autophosphorylation reaction of HK and the forward phosphorelays:

$$\mathrm{HK} \xrightarrow{k_s} \mathrm{HKp} \qquad \mathrm{HKp} + \mathrm{REC} \xrightarrow{k_2} \mathrm{HK} + \mathrm{RECp}$$

$$\mathrm{RECp} + \mathrm{Hpt} \xrightarrow{k_3} \mathrm{REC} + \mathrm{Hptp} \qquad \mathrm{Hptp} + \mathrm{RR} \xrightarrow{k_4} \mathrm{Hpt} + \mathrm{RRp}.$$

We study extensions of this minimal reaction mechanism obtained by adding reverse phosphotransfer reactions involving HK, REC, Hpt and RR, as well as hydrolysis reactions of RECp and RRp:

$$\mathrm{HK} + \mathrm{RECp} \xrightarrow{k_{2r}} \mathrm{HKp} + \mathrm{REC} \qquad \mathrm{RECp} \xrightarrow{k_{h1}} \mathrm{REC}$$

$$\mathrm{REC} + \mathrm{Hptp} \xrightarrow{k_{3r}} \mathrm{RECp} + \mathrm{Hpt} \qquad \mathrm{RRp} \xrightarrow{k_{h2}} \mathrm{RR}$$

$$\mathrm{Hpt} + \mathrm{RRp} \xrightarrow{k_{4r}} \mathrm{Hptp} + \mathrm{RR}.$$

By setting some of the rate constants k_{*r} or k_{h*} to zero, we obtain 32 different topologies involving different combinations of reverse phosphorelay and hydrolysis reactions (Table 1 and Table S1). The minimal set of reactions is always part of the system, meaning that $k_2, k_3, k_4, k_s \neq 0$ for all topologies.

Ordinary differential equations. We model the protein concentrations in the system using ordinary differential equations (ODEs). To simplify the notation, we define:

$$x_1 = [\mathrm{HK}] \quad x_2 = [\mathrm{HKp}] \quad x_3 = [\mathrm{REC}] \quad x_4 = [\mathrm{RECp}]$$
$$x_5 = [\mathrm{Hpt}] \quad x_6 = [\mathrm{Hptp}] \quad x_7 = [\mathrm{RR}] \quad x_8 = [\mathrm{RRp}].$$

The dynamics of the concentrations in time is modeled with a system of ODEs:

$$\begin{aligned}
\dot{x}_1 &= -k_s x_1 - k_{2r} x_1 x_4 + k_2 x_2 x_3 \\
\dot{x}_2 &= k_s x_1 + k_{2r} x_1 x_4 - k_2 x_2 x_3 \\
\dot{x}_3 &= -k_2 x_2 x_3 + k_{h1} x_4 + k_{2r} x_1 x_4 + k_3 x_4 x_5 - k_{3r} x_3 x_6 \\
\dot{x}_4 &= k_2 x_2 x_3 - k_{h1} x_4 - k_{2r} x_1 x_4 - k_3 x_4 x_5 + k_{3r} x_3 x_6 \\
\dot{x}_5 &= -k_3 x_4 x_5 + k_{3r} x_3 x_6 + k_4 x_6 x_7 - k_{4r} x_5 x_8 \\
\dot{x}_6 &= k_3 x_4 x_5 - k_{3r} x_3 x_6 - k_4 x_6 x_7 + k_{4r} x_5 x_8 \\
\dot{x}_7 &= -k_4 x_6 x_7 + k_{h2} x_8 + k_{4r} x_5 x_8 \\
\dot{x}_8 &= k_4 x_6 x_7 - k_{h2} x_8 - k_{4r} x_5 x_8.
\end{aligned} \tag{S1}$$

We note that

$$\dot{x}_1 + \dot{x}_2 = 0, \qquad \dot{x}_3 + \dot{x}_4 = 0, \qquad \dot{x}_5 + \dot{x}_6 = 0, \qquad \dot{x}_7 + \dot{x}_8 = 0.$$

As a consequence $x_i + x_{i+1}$ is constant for $i = 1, 3, 5, 7$ and the system has four conserved amounts:

$$\mathrm{HK_{tot}} = x_1 + x_2, \quad \mathrm{REC_{tot}} = x_3 + x_4, \quad \mathrm{Hpt_{tot}} = x_5 + x_6, \quad \mathrm{RR_{tot}} = x_7 + x_8,$$

where $\mathrm{HK_{tot}}, \mathrm{REC_{tot}}, \mathrm{Hpt_{tot}}$, and $\mathrm{RR_{tot}}$ are positive constants given by the initial concentrations of the system. To ease the writing, we change the notation to

$$\overline{H} := \mathrm{HK_{tot}}, \quad \overline{C} := \mathrm{REC_{tot}}, \quad \overline{T} := \mathrm{Hpt_{tot}}, \quad \overline{R} := \mathrm{RR_{tot}}.$$

Steady-state equations. The steady states of the system are found by setting the derivatives, $\dot{x}_i$, of the concentrations to zero, that is, $\dot{x}_i = 0$. By equating the right-hand side of the ODEs to zero we obtain a system of polynomial equations in the concentrations x_i.

Due to the existence of conserved amounts, some equations are redundant. For instance, the first and second steady-state equations are

$$0 = -k_s x_1 - k_{2r} x_1 x_4 + k_2 x_2 x_3, \qquad 0 = k_s x_1 + k_{2r} x_1 x_4 - k_2 x_2 x_3.$$

One equation is minus the other, and hence, if one of them is fulfilled then so is the other. This happens because $x_1 + x_2$ is conserved. In total, four of the steady-state equations are redundant and must be replaced by the conservation equations. The steady states of the system are thus given as the solutions to the following system of equations:

$$\begin{aligned}
\overline{H} &= x_1 + x_2 && \text{(S2)} & 0 &= k_s x_1 + k_{2r} x_1 x_4 - k_2 x_2 x_3 && \text{(S6)}\\
\overline{C} &= x_3 + x_4 && \text{(S3)} & 0 &= k_2 x_2 x_3 - k_{h1} x_4 - k_{2r} x_1 x_4 - k_3 x_4 x_5 + k_{3r} x_3 x_6 && \text{(S7)}\\
\overline{T} &= x_5 + x_6 && \text{(S4)} & 0 &= k_3 x_4 x_5 - k_{3r} x_3 x_6 - k_4 x_6 x_7 + k_{4r} x_5 x_8 && \text{(S8)}\\
\overline{R} &= x_7 + x_8 && \text{(S5)} & 0 &= k_4 x_6 x_7 - k_{h2} x_8 - k_{4r} x_5 x_8. && \text{(S9)}
\end{aligned}$$

Only solutions with non-negative solutions are meaningful, that is, all concentrations must be positive or zero. Therefore, a steady state will always refer to a non-negative steady state.

If the rate constants and the total amounts are known, then we solve this system of equations to find the steady states. This can be done using mathematical software such as Matlab, Maple, or Mathematica.

Rearrangement of the steady-state equations. The set of equations (S2)-(S9) can be replaced by another system of equations that is easier to interpret. We change equations (S6)-(S9) by linear combinations of them. This process does not change the set of solutions to the system. Specifically, we replace:

(S8) by (S8)+(S9), (S7) by (S7)+(S8)+(S9), (S6) by (S6)+(S7)+(S8)+(S9),

and leave (S9) as it is. This results in the following equivalent system of equations:

$$\begin{aligned}
\overline{H} &= x_1 + x_2 && \text{(S10)} & 0 &= k_s x_1 - k_{h1} x_4 - k_{h2} x_8 && \text{(S14)}\\
\overline{C} &= x_3 + x_4 && \text{(S11)} & 0 &= k_2 x_2 x_3 - k_{h1} x_4 - k_{2r} x_1 x_4 - k_{h2} x_8 && \text{(S15)}\\
\overline{T} &= x_5 + x_6 && \text{(S12)} & 0 &= k_3 x_4 x_5 - k_{3r} x_3 x_6 - k_{h2} x_8 && \text{(S16)}\\
\overline{R} &= x_7 + x_8 && \text{(S13)} & 0 &= k_4 x_6 x_7 - k_{h2} x_8 - k_{4r} x_5 x_8, && \text{(S17)}
\end{aligned}$$

where (S2)-(S5) are identical to (S10)-(S13), and (S17) is identical to (S9).

1.2 Steady-state relations and signal-response curve

We proceed to find an analytical expression of the signal-response curve. In this system, the *signal* is taken to be the value of the rate constant k_s, and the *response* is the steady-state value of the phosphorylated response regulator (x_8) corresponding to k_s (with all the other rate constants and total amounts fixed).

In order to find an analytical relation between k_s and x_8, we establish an analytical relation between each concentration x_i and x_8 at steady state. For some topologies, however, the signal-response curve is constant. We start by discussing when this is the case and then proceed to study the remaining topologies.

Topologies with constant signal-response curve. In the following cases, the signal-response curve is constant and equals $\mathrm{RR}_{\mathrm{tot}}$ (i.e. $x_8 = \overline{R}$ at steady state, for any non-zero value of k_s). Hence, the signal-response curve cannot have hyperbolic or sigmoidal shape, or show ultrasensitivity in these cases:

- $k_{h1} = k_{h2} = 0$.
- $k_{h2} = k_{3r} = 0$.
- $k_{h2} = k_{4r} = 0$.

The topologies that belong to at least one of the cases above are: **topologies 9-13, 15** and **topologies 17-24**. All the other topologies have non-constant signal-response curves and all concentrations at steady state are non-zero. In particular, if $k_{h2} \neq 0$, that is, if there is hydrolysis at RRp, then the signal-response curve is not constant.

These claims are proven in Appendix A.1.

Steady-state analytical relations. We have expressed each concentration x_i at steady state as a function of x_8. This implies that if the value of x_8 at steady state is known, then so are the values of $x_1, \dots, x_7$.

We assume that if $k_{h2} = 0$ then either $k_{h1} \neq 0$, $k_{3r} \neq 0$ or $k_{4r} \neq 0$. That is, we consider only the cases for which the signal-response curve is not constant. In particular, there are no zero concentrations at steady state and hence all steady states must be positive at each concentration.

The following lists an iterative way to find the steady-state values of all variables, once the value of x_8 is known. These expressions are derived in Appendix A.2 using equations (S10)-(S13) and (S15)-(S17).

(S18)

Expression	Behavior as function of x_8
$x_7 = \overline{R} - x_8$	x_7 decreases in x_8
$x_5 = \frac{k_4\overline{T}x_7 - k_{h2}x_8}{k_4x_7 + k_{4r}x_8}$	x_5 decreases in x_8
$x_6 = \overline{T} - x_5$	x_6 increases in x_8
$x_3 = \frac{k_3\overline{C}x_5 - k_{h2}x_8}{k_3x_5 + k_{3r}x_6}$	x_3 decreases in x_8
$x_4 = \overline{C} - x_3$	x_4 increases in x_8
$x_1 = \frac{k_2\overline{H}x_3 - k_{h1}x_4 - k_{h2}x_8}{k_2x_3 + k_{2r}x_4}$	x_1 decreases in x_8
$x_2 = \overline{H} - x_1$	x_2 increases in x_8

By plugging in iteratively the explicit expressions in x_8 we obtain that each variable is expressed at steady state as the quotient of two polynomials in x_8. The steady-state values are all positive if and only if x_8 belongs to the interval $I = (0, \alpha)$, where α is the first positive root of the degree-2 polynomial:

$$q_2(x_8) := a_2 x_8^2 + a_1 x_8 + a_0 \tag{S19}$$

with

$$\begin{aligned}
a_2 &= k_{h2}(k_4 - k_{4r})(k_{h1} + k_2\overline{H}) + k_{h2}^2(k_3 - k_{3r}) + k_{h2}(k_3k_4 - k_{3r}k_{4r})\overline{T} \\
a_1 &= -k_{h1}(k_{3r}\overline{C}(k_{h2} + k_{4r}\overline{T}) + k_{h2}k_4\overline{R}) - k_{h2}(k_2\overline{H}(k_4\overline{R} + k_3\overline{C}) + k_3k_4\overline{TR}) - k_2k_3k_4\overline{HCT} \\
a_0 &= k_2k_3k_4\overline{HCTR}
\end{aligned}$$

(see Appendix A.2 for a proof).

Signal-response expression. Using the remaining steady-state equation, (S14), we express k_s in terms of x_8 (see Appendix A.3):

$$k_s = \frac{k_{h1}x_4 + k_{h2}x_8}{x_1}.$$

Since x_4 increases in x_8 and x_1 decreases in x_8, it follows that k_s increases in x_8. If we express x_4, x_1 in terms of x_8 using (S18), we obtain that the exact analytical expression relating k_s and x_8 is:

$$k_s = f(x_8) = \frac{x_8 p_1(x_8) p_2(x_8)}{q_1(x_8) q_2(x_8)} \tag{S20}$$

with $q_2(x_8)$ given as in (S19) and

$$\begin{aligned}
q_1(x) &= (k_{h2}(k_3 - k_{3r}) + (k_3k_4 - k_{3r}k_{4r})\overline{T})x - k_3k_4\overline{RT}, \\
p_1(x) &= k_{h2}\big(k_{h1}(k_4 - k_{4r}) + k_{h2}(k_3 - k_{3r}) + (k_3k_4 - k_{3r}k_{4r})\overline{T}\big)x \\
&\quad - k_4k_{h2}(k_{h1} + k_3\overline{T})\overline{R} - k_{3r}k_{h1}(k_{h2} + k_{4r}\overline{T})\overline{C}, \\
p_2(x) &= k_{h2}(k_2 - k_{2r})(k_4 - k_{4r})x^2 + k_2k_3k_4\overline{RCT} + \\
&\quad \big(k_{h2}(\, k_4(k_{2r} - k_2)\overline{R} + (k_{2r}k_{3r} - k_2k_3)\overline{C}\,) + (k_{2r}k_{3r}k_{4r} - k_2k_3k_4)\overline{CT}\big)x.
\end{aligned}$$

This function is well defined for x_8 is in $I = (0, \alpha)$, that is, it is positive and continuous. When x_8 approaches α (the upper bound of the interval I), then k_s tends to infinity (the denominator of f tends to zero). Therefore, the image of f is the interval $(0, +\infty)$. Further the function f can be differentiably extended at zero such that $f(0) = 0$.

Given a rate constant k_s, there is a unique value of x_8 for which $f(x_8) = k_s$. This value is the steady-state value of x_8 corresponding to k_s, and the other steady states are found using (S18).

Properties of the signal-response curve. We let φ denote the inverse of f, that is,

$$\varphi(k_s) = x_8 \qquad \text{if } k_s = f(x_8).$$

Using the Inverse Function Theorem, the signal-response function φ is continuous and differentiable in $[0, +\infty)$. We do not have an analytical expression for φ, only of its inverse. However, most of the information required from φ can be retrieved from f:

(i) The function φ is increasing.

(ii) α is the maximal value of the response, x_8. When the activation rate k_s tends to infinity, then x_8 approaches α.

(iii) The derivative of φ at a point $k_s = k$ equals $\varphi'(k) = 1/f'(x_8)$ for $x_8 = \varphi(k)$.

(iv) The second derivative of φ at a point $k_s = k$ equals $\varphi''(k) = -f''(x_8)/f'(x_8)^3$ for $x_8 = \varphi(k)$.

For example, the derivative of the signal-response curve φ at zero is:

$$\frac{k_3 k_4 \overline{HRT}}{k_4 k_{h2}(k_{h1} + k_3\overline{T})\overline{R} + k_{3r} k_{h1}(k_{h2} + k_{4r}\overline{T})\overline{C}}. \tag{S21}$$

Since f is an increasing function in I, we have that $\varphi'(k) > 0$ for all $k \geq 0$ and the sign of the second derivative of φ at k is *minus* the sign of the second derivative of f at $\varphi(k)$.

Practical considerations.

(v) The signal-response curve is plotted by generating points $(f(x_8), x_8)$.

(vi) The maximal response is easily computed as the first positive root of $q_2(x)$, which is a degree-2 polynomial.

(vii) Given k_s, the steady-state value of x_8 is the first positive zero of the polynomial

$$k_s q_1(x_8) q_2(x_8) - x_8 p_1(x_8) p_2(x_8).$$

The other steady-state values are obtained from x_8 and (S18).

1.3 Hyperbolic and sigmoidal signal-response curves

Second derivative at zero. A function $g(x)$ that increases at a slower and slower rate is called hyperbolic, that is the derivative $g'(x)$ of g is decreasing or, alternatively, the second derivative is negative, $g''(x) < 0$. A function $g(x)$ that initially increases at a faster and faster rate and then slows down is called sigmoidal, that is, $g'(x)$ is initially increasing then decreasing or, alternatively, $g''(x)$ is first positive and then becomes negative.

It is difficult in general to establish if a curve is sigmoidal or hyperbolic (or none of these) and we use a simple test to indicate if φ is sigmoidal or hyperbolic. If the second derivative of φ at zero is positive, then the first derivative grows indicating that the curve will likely be sigmoidal. If, on the contrary, the second derivative of φ at zero is negative, then the curve is likely to be hyperbolic. This test is a good indicator of the shape of φ, but note that the test only considers the behavior near zero.

We have observed a perfect overlap between the classification obtained using the sign of the second derivative only at zero and the classification obtained by checking whether the second derivative of the entire signal-response curve changes sign (see *Methods*). This supports that the classification based on the sign of the second derivative at zero is reasonable.

We compute $\varphi''(0)$ using item (iv) above and $\varphi(0) = 0$. Computations are performed in Mathematica. The sign of $\varphi''(0)$ agrees with the sign of:

$$\begin{aligned} S = &- k_{h1}(k_{4r}\overline{T} + k_{h2})\Big(k_{3r}(k_2 k_3 k_4 - k_2 k_{3r} k_{4r} + k_{2r} k_{3r} k_{4r})\overline{CT} + k_4 k_{2r} k_{3r} k_{h2}\overline{R} \\ &\quad + k_{h2}(k_2 k_3 - k_2 k_{3r} + k_{2r} k_{3r})(k_4\overline{R} + k_{3r}\overline{C})\Big)\overline{HC} \\ &- k_{2r} k_4 k_{h2}(k_3 k_{3r}(k_{4r}\overline{T} + k_{h2})\overline{CT} + k_4 k_{h2}(k_3\overline{T} + k_{h1})\overline{R})\overline{HR} \\ &- (k_{3r} k_{h1}(k_{4r}\overline{T} + k_{h2})\overline{C} + k_4 k_{h2}(k_3\overline{T} + k_{h1})\overline{R})^2. \end{aligned} \tag{S22}$$

If $S > 0$, then the signal-response curve is classified as sigmoidal. If $S < 0$, then the signal-response curve is classified as hyperbolic. If the blue terms are all positive, then the curve is hyperbolic and hence only if some of the highlighted blue terms are negative can $S > 0$. Note that the negative terms in S are multiplied by k_{h1}. Therefore, *necessary* conditions for φ to be sigmoidal are

$$k_{h1} > 0 \quad \text{and} \quad (k_2k_3k_4 - k_2k_{3r}k_{4r} + k_{2r}k_{3r}k_{4r} < 0 \quad \text{or} \quad k_2k_3 - k_2k_{3r} + k_{2r}k_{3r} < 0).$$

In particular, if $k_{3r} = 0$ then sigmoidality cannot occur. By inspecting in detail the two blue terms, we see that

$$k_2k_3 - k_2k_{3r} + k_{2r}k_{3r} = k_2(k_3 - k_{3r}) + k_{2r}k_{3r} = k_2k_3 + (k_{2r} - k_2)k_{3r},$$
$$k_2k_3k_4 - k_2k_{3r}k_{4r} + k_{2r}k_{3r}k_{4r} = k_2(k_3k_4 - k_{3r}k_{4r}) + k_{2r}k_{3r}k_{4r} = k_2k_3k_4 + (k_{2r} - k_2)k_{3r}k_{4r}.$$

We conclude that necessary conditions for φ to be sigmoidal are:

$$k_{h1} > 0 \quad \text{and} \quad k_2 > k_{2r} \quad \text{and} \quad (k_{3r} > k_3 \quad \text{or} \quad k_{3r}k_{4r} > k_3k_4). \tag{S23}$$

Further, we conclude the following from an analysis of the expression of S:

- If $\overline{H}$ or k_2 are very small, then S is negative and hence φ is hyperbolic.
- If $k_{h2} = 0$, then the sign of S agrees with the sign of

  $$\overline{H}(k_2k_3k_4 - k_2k_{3r}k_{4r} + k_{2r}k_{3r}k_{4r}) + k_{3r}k_{4r}k_{h1}.$$

  In this case, necessary conditions for φ to be sigmoidal are:

  $$k_{h1} \neq 0 \quad \text{and} \quad k_2 > k_{2r} \quad \text{and} \quad k_{3r}k_{4r} > k_3k_4.$$

Hyperbolic curves. We have also shown (see Appendix A.4) that if:

$$k_2 - k_{2r} > 0, \qquad \text{and} \qquad (k_3 - k_{3r})k_{h2}(k_4\overline{R} + k_{3r}\overline{C}) + k_{3r}(k_3k_4 - k_{3r}k_{4r})\overline{CT} > 0, \tag{S24}$$

then the second derivative of φ strictly decreases over I and hence the curve is hyperbolic (that is, not only the second derivative at zero indicates so). These two inequalities are fulfilled if

$$k_2 > k_{2r}, \qquad k_3 > k_{3r}, \qquad k_4 > k_{4r}.$$

That is, if the phosphorelay rate constants are larger than their reverse counterparts then the curve is hyperbolic. However, the curve can be hyperbolic without these inequalities being fulfilled.

Tuning by varying total amounts. By expressing the term S in (S22) as a polynomial in one of the total amounts, we can observe that for some parameter values, variation of the total amounts can change the system's response from sigmoidal (S positive) to hyperbolic (S negative and *vice versa*. This fact is summarized in the following table:

Total amount	Degree	Independent coefficient	Leading coefficient	Sign of S
$\overline{H}$	1	negative	positive for some parameters	$S < 0$ for $\overline{H}$ small, $S > 0$ for $\overline{H}$ large
$\overline{C}$	2	negative	positive for some parameters	$S < 0$ for $\overline{C}$ small, $S > 0$ for $\overline{C}$ large
$\overline{T}$	2	positive for some parameters	negative	$S > 0$ for $\overline{T}$ small, $S < 0$ for $\overline{C}$ large
$\overline{R}$	2	positive for some parameters	negative	$S > 0$ for $\overline{R}$ small, $S < 0$ for $\overline{C}$ large

The results of the last column in the table hold for any choice of parameters that make the leading or independent coefficient (depending on the total amount) positive.

1.4 Examples

We consider a specific example with rate constants and total amounts given by

$$k_{h1} = 0.5 \qquad k_{h2} = 1 \qquad k_2 = 0.1 \qquad k_4 = 1 \qquad k_{2r} = 1 \qquad k_{3r} = 1$$
$$k_{4r} = 0.5 \qquad k_3 = 0.3 \qquad \overline{H} = 1 \qquad \overline{R} = 10 \qquad \overline{T} = 10 \qquad \overline{C} = 100.$$

The signal-response function f given in (S20) is:

$$k_s = \frac{x_8(505 - 16.25x_8)(2000 + 389x_8 + 0.45x_8^2)}{(-200 + 16x_8)(2000 - 726x_8 + 16.3x_8^2)},$$

such that

$$q_2(x_8) = 2000 - 726x_8 + 16.3x_8^2.$$

The first positive real root of q_2 is $\alpha = 2.95024$. The graph of f in $[0, \alpha)$ is shown in Figure S4(A). If k_s is given, then the anti-image of k_s in this graph is the steady-state value of x_8 and is found as shown in Figure S4(B). Since the curve is strictly increasing, there is one and only one anti-image. We see from the graph that as k_s becomes large, x_8 approaches α, but the value of x_8 can never exceed α. That is, α is the upper bound of the response x_8. The graph of the signal-response curve φ is obtained by reversing the axes (Figure S4(C)). Therefore, when k_s tends to infinity, x_8 approaches the maximal response α.

1.5 Model with intermediates

Reactions. We extend the model given in Subsection 1.1 to incorporate the formation of intermediate complexes at the phosphotransfer reactions. That is, the model extended with intermediates consist of the reactions

$$\mathrm{HK} \xrightarrow{k_s} \mathrm{HKp} \qquad \mathrm{HKp} + \mathrm{REC} \underset{k_{2ar}}{\overset{k_{2a}}{\rightleftarrows}} \mathrm{Y}_1 \underset{k_{2br}}{\overset{k_{2b}}{\rightleftarrows}} \mathrm{HK} + \mathrm{RECp}$$
$$\mathrm{RECp} \xrightarrow{k_{h1}} \mathrm{REC} \qquad \mathrm{RECp} + \mathrm{Hpt} \underset{k_{3ar}}{\overset{k_{3a}}{\rightleftarrows}} \mathrm{Y}_2 \underset{k_{3br}}{\overset{k_{3b}}{\rightleftarrows}} \mathrm{REC} + \mathrm{Hptp}$$
$$\mathrm{RRp} \xrightarrow{k_{h2}} \mathrm{RR} \qquad \mathrm{Hptp} + \mathrm{RR} \underset{k_{4ar}}{\overset{k_{4a}}{\rightleftarrows}} \mathrm{Y}_3 \underset{k_{4br}}{\overset{k_{4b}}{\rightleftarrows}} \mathrm{Hpt} + \mathrm{RRp}.$$

By setting some of the rate constants k_{*r} or k_{h*} to zero, we obtain different topologies involving different combinations of reverse phosphorelay and hydrolysis reactions as before.

Ordinary differential equations. We model the protein concentrations in the system using ordinary differential equations (ODEs). To simplify the notation, we define:

$$x_1 = [\mathrm{HK}] \qquad x_2 = [\mathrm{HKp}] \qquad x_3 = [\mathrm{REC}] \qquad x_4 = [\mathrm{RECp}]$$
$$x_5 = [\mathrm{Hpt}] \qquad x_6 = [\mathrm{Hptp}] \qquad x_7 = [\mathrm{RR}] \qquad x_8 = [\mathrm{RRp}]$$
$$x_9 = [Y_1] \qquad x_{10} = [Y_2] \qquad x_{11} = [Y_3].$$

The dynamics of the concentrations in time is modeled with a system of ODEs:

$$\begin{aligned}
\dot{x}_1 &= -k_s x_1 - k_{2br} x_1 x_4 + k_{2b} x_9 \\
\dot{x}_2 &= k_s x_1 + k_{2ar} x_9 - k_{2a} x_2 x_3 \\
\dot{x}_3 &= -k_{2a} x_2 x_3 + k_{h1} x_4 + k_{2ar} x_9 + k_{3b} x_{10} - k_{3br} x_3 x_6 \\
\dot{x}_4 &= k_{2b} x_9 - k_{h1} x_4 - k_{2br} x_1 x_4 - k_{3a} x_4 x_5 + k_{3ar} x_{10} \\
\dot{x}_5 &= -k_{3a} x_4 x_5 + k_{3ar} x_{10} + k_{4b} x_{11} - k_{4br} x_5 x_8 \\
\dot{x}_6 &= k_{3b} x_{10} - k_{3br} x_3 x_6 - k_{4a} x_6 x_7 + k_{4ar} x_{11} \\
\dot{x}_7 &= -k_{4a} x_6 x_7 + k_{h2} x_8 + k_{4ar} x_{11} \\
\dot{x}_8 &= k_{4b} x_{11} - k_{h2} x_8 - k_{4br} x_5 x_8 \\
\dot{x}_9 &= k_{2a} x_2 x_3 - k_{2ar} x_9 + k_{2br} x_1 x_4 - k_{2b} x_9 \\
\dot{x}_{10} &= k_{3a} x_4 x_5 - k_{3ar} x_{10} - k_{3b} x_{10} + k_{3br} x_3 x_6 \\
\dot{x}_{11} &= k_{4a} x_6 x_7 - k_{4ar} x_{11} + k_{4br} x_5 x_8 - k_{4b} x_{11}.
\end{aligned}$$

The system has four conserved amounts:

$$\mathrm{HK}_{\mathrm{tot}} = x_1 + x_2 + x_9, \quad \mathrm{REC}_{\mathrm{tot}} = x_3 + x_4 + x_9 + x_{10}, \quad \mathrm{Hpt}_{\mathrm{tot}} = x_5 + x_6 + x_{10} + x_{11}, \quad \mathrm{RR}_{\mathrm{tot}} = x_7 + x_8 + x_{11},$$

where $\mathrm{HK}_{\mathrm{tot}}, \mathrm{REC}_{\mathrm{tot}}, \mathrm{Hpt}_{\mathrm{tot}}$, and $\mathrm{RR}_{\mathrm{tot}}$ are positive constants given by the initial concentrations of the system. As before, we write

$$\overline{H} := \mathrm{HK}_{\mathrm{tot}}, \quad \overline{C} := \mathrm{REC}_{\mathrm{tot}}, \quad \overline{T} := \mathrm{Hpt}_{\mathrm{tot}}, \quad \overline{R} := \mathrm{RR}_{\mathrm{tot}}.$$

Steady-state equations. We proceed as in the monofunctional case and conclude that the steady states of the system are given as the solutions to the following system of equations:

$$\begin{aligned}
\overline{H} &= x_1 + x_2 + x_9 \\
\overline{C} &= x_3 + x_4 + x_9 + x_{10} \\
\overline{T} &= x_5 + x_6 + x_{10} + x_{11} \\
\overline{R} &= x_7 + x_8 + x_{11}
\end{aligned}
\qquad
\begin{aligned}
0 &= -k_s x_1 - k_{2br} x_1 x_4 + k_{2b} x_9 \\
0 &= -k_{2a} x_2 x_3 + k_{h1} x_4 + k_{2ar} x_9 + k_{3b} x_{10} - k_{3br} x_3 x_6 \\
0 &= k_{2b} x_9 - k_{h1} x_4 - k_{2br} x_1 x_4 - k_{3a} x_4 x_5 + k_{3ar} x_{10} \\
0 &= -k_{3a} x_4 x_5 + k_{3ar} x_{10} + k_{4b} x_{11} - k_{4br} x_5 x_8 \\
0 &= k_{3b} x_{10} - k_{3br} x_3 x_6 - k_{4a} x_6 x_7 + k_{4ar} x_{11} \\
0 &= -k_{4a} x_6 x_7 + k_{h2} x_8 + k_{4ar} x_{11} \\
0 &= k_{4b} x_{11} - k_{h2} x_8 - k_{4br} x_5 x_8
\end{aligned}$$

Hyperbolic vs. sigmoidal. It is not so straightforward in this case to obtain an analytical description of the signal-response curve. Therefore, we adopt a direct route to the computation of the sign of the second derivative at zero.

When $k_s = 0$, then the steady state of the system equals

$$(x_1, \dots, x_{11}) = (\overline{H}, 0, \overline{C}, 0, \overline{T}, 0, \overline{R}, 0, 0, 0, 0).$$

We want to find the derivative of the response x_8 with respect to k_s at $k_s = 0$ at steady state. To this end, we do the following steps:

1. We take the derivative with respect to k_s of both sides of the steady-state equations. We obtain new equations, where $p_i = \frac{\partial x_i}{\partial k_s}$:

$$
\begin{aligned}
0 &= -x_1 - k_s p_1 - k_{2br} p_1 x_4 - k_{2br} x_1 p_4 + k_{2b} p_9,\\
0 &= -k_{2a} p_2 x_3 - k_{2a} x_2 p_3 + k_{2ar} p_9 - k_{3br} p_3 x_6 - k_{3br} x_3 p_6 + k_{3b} p_{10} + k_{h1} p_4,\\
0 &= -k_{2br} p_1 x_4 - k_{2br} x_1 p_4 + k_{2b} p_9 - k_{3a} p_4 x_5 - k_{3a} x_4 p_5 + k_{3ar} p_{10} - k_{h1} p_4,\\
0 &= -k_{3a} p_4 x_5 - k_{3a} x_4 p_5 + k_{3ar} p_{10} - k_{4br} p_5 x_8 - k_{4br} x_5 p_8 + k_{4b} p_{11},\\
0 &= -k_{3br} p_3 x_6 - k_{3br} x_3 p_6 + k_{3b} p_{10} - k_{4a} p_6 x_7 - k_{4a} x_6 p_7 + k_{4ar} p_{11},\\
0 &= -k_{4a} p_6 x_7 - k_{4a} x_6 p_7 + k_{4ar} p_{11} + k_{h2} p_8,\\
0 &= -k_{4br} p_5 x_8 - k_{4br} x_5 p_8 + k_{4b} p_{11} - k_{h2} p_8,\\
0 &= p_1 + p_2 + p_9,\\
0 &= p_3 + p_4 + p_9 + p_{10},\\
0 &= p_5 + p_6 + p_{10} + p_{11},\\
0 &= p_7 + p_8 + p_{11}.
\end{aligned}
$$

2. We substitute, in the equations above, the steady-state value when $k_s = 0$ and obtain:

$$
\begin{aligned}
0 &= -\overline{H} - k_s p_1(0) - k_{2br} p_1(0) - k_{2br} \overline{H} p_4(0) + k_{2b} p_9(0),\\
0 &= -k_{2a} \overline{C} p_2(0) - k_{2a} p_3(0) + k_{2ar} p_9(0) - k_{3br} p_3(0) - k_{3br} \overline{C} p_6(0) + k_{3b} p_{10}(0) + k_{h1} p_4(0),\\
0 &= -k_{2br} p_1(0) - k_{2br} \overline{H} p_4(0) + k_{2b} p_9(0) - k_{3a} \overline{T} p_4(0) - k_{3a} p_5(0) + k_{3ar} p_{10}(0) - k_{h1} p_4(0),\\
0 &= -k_{3a} \overline{T} p_4(0) - k_{3a} p_5(0) + k_{3ar} p_{10}(0) - k_{4br} p_5(0) - k_{4br} \overline{T} p_8(0) + k_{4b} p_{11}(0),\\
0 &= -k_{3br} p_3(0) - k_{3br} \overline{C} p_6(0) + k_{3b} p_{10}(0) - k_{4a} \overline{R} p_6(0) - k_{4a} p_7(0) + k_{4ar} p_{11}(0),\\
0 &= -k_{4a} \overline{R} p_6(0) - k_{4a} p_7(0) + k_{4ar} p_{11}(0) + k_{h2} p_8(0),\\
0 &= -k_{4br} p_5(0) - k_{4br} \overline{T} p_8(0) + k_{4b} p_{11}(0) - k_{h2} p_8(0),\\
0 &= p_1(0) + p_2(0) + p_9(0),\\
0 &= p_3(0) + p_4(0) + p_9(0) + p_{10}(0),\\
0 &= p_5(0) + p_6(0) + p_{10}(0) + p_{11}(0),\\
0 &= p_7(0) + p_8(0) + p_{11}(0).
\end{aligned}
$$

This system is linear in $p_1(0), \dots, p_{11}(0)$ and hence the derivatives of x_i at $k_s = 0$ can be found by solving the system. We have solved it using Maple. In particular, we have obtained that

$$
p_8(0) = \frac{k_{4a} k_{3b} k_{3a} k_{4b} \overline{HRT}}{k_{h2} k_{4a} k_{3b} k_{4b} \overline{R}(k_{h1} + k_{3a}\overline{T}) + k_{h1} k_{3ar}(k_{h2} k_{4ar}(k_{3br}\overline{C} + k_{4a}\overline{R}) + k_{3br}\overline{C}(k_{h2} k_{4b} + k_{4br} k_{4ar} \overline{T}))} \tag{S25}
$$

This is the derivative of the signal-response curve at $k_s = 0$.

3. We repeat the steps above one more time: we compute the derivative with respect to k_s of the above equations (step 1). We evaluate the resulting equations at the steady state for $k_s = 0$ and at $p_i = p_i(0)$. We obtain a linear system in the second derivatives of x_i at $k_s = 0$ which can be solved in Maple. As a result, we obtain the second derivative of x_8 with respect to k_s at $k_s = 0$ as desired.

Before showing what the second derivative of x_8 with respect to k_s at $k_s = 0$ is, it is convenient to introduce new parameters. For $i = 2, 3, 4$, let

$$k_{iy} = \frac{k_{ia}}{k_{iar} + k_{ib}} \qquad k_{iyr} = \frac{k_{ibr}}{k_{iar} + k_{ib}} \qquad k_i = k_{ib}k_{iy} \qquad k_{ir} = k_{iar}k_{iyr}.$$

For an interpretation of these constants see below. In particular, we take k_i, k_{ir} to be the rates of forward and reverse phosphorylation at each layer.

With these new constants, we obtain that the derivative of x_8 with respect to k_s at $k_s = 0$, that is (S25), becomes

$$p_8(0) = \frac{k_3 k_4 \overline{HRT}}{k_4 k_{h2}(k_{h1} + k_3\overline{T})\overline{R} + k_{3r}k_{h1}(k_{h2} + k_{4r}\overline{T})\overline{C}}. \tag{S26}$$

This expression is identical to the first derivative of x_8 with respect to k_s at $k_s = 0$ for the model without intermediates, as given in (S21).

Similarly, the sign of the second derivative of x_8 with respect to k_s at $k_s = 0$ equals the sign of:

$$\begin{aligned} S_y = S &- CHk_2k_3k_{h1}(k_{4y}(k_{4r}T + k_{h2}) + Tk_4k_{4yr})(Rz_2 + CTz_1) \\ &- CHk_2k_{h1}(z_2 - Tz_1)((Cz_1 + Rk_{h2}k_4)k_{3y} + C(k_{4r}T + k_{h2})k_3k_{3yr}) \\ &- C(k_{h1}z_1(C - H) + z_3)(H(Cz_1 + Rk_{h2}k_4)(k_2k_{2yr} + k_{2r}k_{2y}) + (Ck_{h1}z_1 + z_3)k_{2y}) \end{aligned} \tag{S27}$$

where S is given in (S22) and

$$z_1 := k_{3r}(k_{4r}T + k_{h2}), \quad z_2 := k_{h2}(k_4R + k_{3r}C), \quad z_3 := Rk_{h2}k_4(k_3T + k_{h1}).$$

Recall that the condition for sigmoidality is $S_y > 0$. We have marked in blue the only terms that can cause the term S_y to be positive. Namely, if S is negative (that is, the model without intermediates is hyperbolic), $C > H$ and $z_2 > Tz_1$, then the model with intermediates is hyperbolic as well.

We deduce easily that

- If $k_{h1} = 0$, then sigmoidality cannot occur.
- If $k_{3r} = 0$ then $z_1 = 0$ and $S < 0$, and hence sigmoidality cannot occur.

Interpretation of the new rate constants. The rate constants k_{iy}, k_{iyr} are the reciprocal of the Michaelis-Menten constants of each intermediate Y_i in each direction. These are the coefficients of the expression in $x_1, \ldots, x_8$ obtained by imposing $\dot{x}_9 = \dot{x}_{10} = \dot{x}_{11} = 0$ and solving for x_9, x_{10}, x_{11}. In particular, at steady state we have:

$$\begin{aligned} x_9 &= \frac{k_{2a}}{k_{2ar} + k_{2b}}x_2x_3 + \frac{k_{2br}}{k_{2ar} + k_{2b}}x_1x_4 = k_{2y}x_2x_3 + k_{2yr}x_1x_4 \\ x_{10} &= \frac{k_{3a}}{k_{3ar} + k_{3b}}x_4x_5 + \frac{k_{3br}}{k_{3ar} + k_{3b}}x_3x_6 = k_{3y}x_4x_5 + k_{3yr}x_3x_6 \\ x_{11} &= \frac{k_{4a}}{k_{4ar} + k_{4b}}x_6x_7 + \frac{k_{4br}}{k_{4ar} + k_{4b}}x_5x_8 = k_{4y}x_6x_7 + k_{4yr}x_5x_8. \end{aligned}$$

If we plug these values into the ODEs $\dot{x}_i$, $i = 1, \ldots, 8$, we obtain a mass-action system for the model without intermediates with rate constants $k_i = k_{ib}k_{iy}$ and $k_{ir} = k_{iar}k_{iyr}$.

1.6 Model with production and degradation

We investigate if the conditions for sigmoidality are altered by the introduction of production and degradation in the model.

Reactions, equations and steady states. We consider the system with reactions as described in Subsection 1.1, together with degradation reactions for all species

$$\mathrm{HK} \xrightarrow{k_{1o}} 0 \qquad \mathrm{HKp} \xrightarrow{k_{2o}} 0 \qquad \mathrm{REC} \xrightarrow{k_{3o}} 0 \qquad \mathrm{RECp} \xrightarrow{k_{4o}} 0$$
$$\mathrm{Hpt} \xrightarrow{k_{5o}} 0 \qquad \mathrm{Hptp} \xrightarrow{k_{6o}} 0 \qquad \mathrm{RR} \xrightarrow{k_{7o}} 0 \qquad \mathrm{RRp} \xrightarrow{k_{8o}} 0$$

and production reactions for the unphosphorylated forms:

$$0 \xrightarrow{k_{1i}} \mathrm{HK} \qquad 0 \xrightarrow{k_{3i}} \mathrm{REC} \qquad 0 \xrightarrow{k_{5i}} \mathrm{Hpt} \qquad 0 \xrightarrow{k_{7i}} \mathrm{RR}$$

We define as usual

$$x_1 = [\mathrm{HK}] \qquad x_2 = [\mathrm{HKp}] \qquad x_3 = [\mathrm{REC}] \qquad x_4 = [\mathrm{RECp}]$$
$$x_5 = [\mathrm{Hpt}] \qquad x_6 = [\mathrm{Hptp}] \qquad x_7 = [\mathrm{RR}] \qquad x_8 = [\mathrm{RRp}].$$

The dynamics of the concentrations in time is modeled with a system of ODEs:

$$\begin{aligned}
\dot{x}_1 &= -k_s x_1 - k_{2r} x_1 x_4 + k_2 x_2 x_3 - k_{1o} x_1 + k_{1i}\\
\dot{x}_2 &= k_s x_1 + k_{2r} x_1 x_4 - k_2 x_2 x_3 - k_{2o} x_2\\
\dot{x}_3 &= -k_2 x_2 x_3 + k_{h1} x_4 + k_{2r} x_1 x_4 + k_3 x_4 x_5 - k_{3r} x_3 x_6 - k_{3o} x_3 + k_{3i}\\
\dot{x}_4 &= k_2 x_2 x_3 - k_{h1} x_4 - k_{2r} x_1 x_4 - k_3 x_4 x_5 + k_{3r} x_3 x_6 - k_{4o} x_4\\
\dot{x}_5 &= -k_3 x_4 x_5 + k_{3r} x_3 x_6 + k_4 x_6 x_7 - k_{4r} x_5 x_8 - k_{5o} x_5 + k_{5i}\\
\dot{x}_6 &= k_3 x_4 x_5 - k_{3r} x_3 x_6 - k_4 x_6 x_7 + k_{4r} x_5 x_8 - k_{6o} x_6\\
\dot{x}_7 &= -k_4 x_6 x_7 + k_{h2} x_8 + k_{4r} x_5 x_8 - k_{7o} x_7 + k_{7i}\\
\dot{x}_8 &= k_4 x_6 x_7 - k_{h2} x_8 - k_{4r} x_5 x_8 - k_{8o} x_8.
\end{aligned}$$

The system does not have any conservation law. Thus, the steady-state equations are given by setting the derivative of the concentration to zero, that is $\dot{x}_i = 0$.

Hyperbolic vs. sigmoidal. The procedure applied to our initial system in Subsection 1.2 to obtain the inverse of the signal-response curve, can be applied here to obtain an analytical expression of the inverse of the signal-response curve. The role of the total amounts $\overline{H}, \overline{C}, \overline{T}$ and $\overline{R}$ is played by the quotients

$$K_1 = \frac{k_{1i}}{k_{1o}}, \qquad K_3 = \frac{k_{3i}}{k_{3o}}, \qquad K_5 = \frac{k_{5i}}{k_{5o}}, \qquad K_7 = \frac{k_{7i}}{k_{7o}}.$$

We do not reproduce the analysis here again. The procedure leads to the derivative of the signal-response curve at zero. Alternatively, we can apply the procedure described in the previous subsection to directly obtain the sign of the second derivative of the signal-response curve at zero, without explicitly computing the signal-response curve.

The expression of the second derivative of the signal-response curve at zero is very large, and hence we only provide here the positive monomials with the aim of determining what architectures can exhibit sigmoidality.

We use the definition of K_1, K_3, K_5, K_7 above, together with

$$K_2 = \frac{k_{2o}}{k_{1o}}, \qquad K_4 = \frac{k_{4o}}{k_{3o}}, \qquad K_6 = \frac{k_{6o}}{k_{5o}}, \qquad K_8 = \frac{k_{8o}}{k_{7o}}.$$

The positive terms that can lead to sigmoidality are then

$$S_{o,pos} = k_2 K_3 K_1 (k_{8o} + k_{4r} K_5 + k_{h2}) \Big(k_{4r} k_{6o} k_3^2 K_5^2 K_6 (k_2 K_3 + k_{2o}) \\ + K_4 K_3 k_2 k_{3r} (k_{h1} + k_{4o}) (k_{4r} K_5 (k_{3r} K_3 + k_{6o}) + (k_{8o} + k_{h2})(k_4 K_7 + k_{3r} K_3 + k_{6o})) \Big).$$

We observe that if $k_{3r} = k_{4r} = 0$ then $S_{o,pos} = 0$ and sigmoidality cannot occur. Contrary to the system without production and degradation, $k_{3r} = 0$ does not guarantee that sigmoidality cannot occur. This is due to the fact that now there is a degradation of Hptp, which plays the role of the hydrolysis k_{h1} at RECp. Therefore, the reverse phosphorelay between layers 3 and 4 can also account for sigmoidality.

In the system with production/degradation reactions, inclusion of intermediates cannot alter steady-state properties such as the existence of sigmoidality. In recent work, we have shown that in reaction schemes that do not give rise to conservation relations, consideration of complex formation does not alter the system properties at steady state [1].

1.7 Model with auto-dephosphorylation at HK

We extend the model given in Subsection 1.1 to incorporate auto-dephosphorylation of HK. That is, we enrich the system with a reaction

$$\text{HKp} \xrightarrow{k_5} \text{HK}.$$

The ODE system modeling the protein concentrations is identical to (S1) except for the expressions for $\dot{x}_1, \dot{x}_2$ that become

$$\dot{x}_1 = -k_s x_1 - k_{2r} x_1 x_4 + k_2 x_2 x_3 + k_5 x_2$$
$$\dot{x}_2 = k_s x_1 + k_{2r} x_1 x_4 - k_2 x_2 x_3 - k_5 x_2.$$

The steps followed in Subsection 1.1 can be applied here as well to obtain an analytical expression of the inverse of the signal-response curve. The sign of the second derivative of the signal-response curve at zero agrees with the sign of

$$S_d := (k_5 + k_2 \overline{C}) S - k_2 k_5 \overline{H} \big((k_3 \overline{T} + k_{2r} \overline{H}) k_4 k_{h2} z_3 \overline{R} + z_1 k_3 k_{2r} \overline{CH} (k_{3r} z_2 \overline{C} + k_4 k_{h2} \overline{R}) + k_{h1} z_3^2 \big)$$

where

$$z_1 = k_{4r} \overline{T} + k_{h2}, \qquad z_2 = k_4 \overline{T} + k_{h2}, \qquad z_3 = k_4 k_{h2} \overline{R} + k_{3r} z_1 \overline{C},$$

and S is given in (S22). We easily see that S_d can only be positive if S is positive. Therefore, the necessary conditions for sigmoidality established in the main text for the simple model are not altered by explicitly modeling auto-dephosphorylation of HK.

1.8 Model with auto-dephosphorylation at Hpt

We extend the model given in Subsection 1.1 to incorporate auto-dephosphorylation of Hpt. That is, we enrich the system with a reaction

$$\mathrm{Hptp} \xrightarrow{\mathrm{k_5}} \mathrm{Hpt}.$$

We applied the steps described in Subsection 1.5 to obtain an expression of the sign of the second derivative of the signal-response curve at zero in terms of the rate constants and total amounts. The sign is given by

$$\begin{aligned} S_3 =& S - k_5 z_1 \Big(z_1 z_2 (k_{h1} k_{3r} \overline{C} + k_3 k_5 \overline{T} + (k_{2r}\overline{H} + k_{h1})(k_{3r}\overline{C} + k_5)) + 2\, k_4 k_{h2} z_2 (\overline{H} k_{2r} + z_2)\overline{R} \\ &+ \overline{CH}(k_2 k_3^2 (k_4 - k_{4r})\overline{T}^2 + (k_2 k_3 k_4 - k_2 k_{3r} k_{4r} + k_{2r} k_{3r} k_{4r}) k_{h1} \overline{T} \\ &+ k_{h1} k_{h2} (k_2 k_3 - k_2 k_{3r} + k_{2r} k_{3r})) \Big) \end{aligned}$$

where

$$z_1 = k_{4r}\overline{T} + k_{h2}, \qquad z_2 = k_3 \overline{T} + k_{h1}$$

and S is given in (S22). The terms highlighted in blue are the terms that can possibly contribute to S_3 being positive. The last two highlighted terms are also highlighted in S in (S22) and further, they are multiplied by k_{h1}.

When k_5 is set to zero, the sign of the second derivative of the signal-response curve at zero agrees with the corresponding sign for the model without auto-dephosphorylation at Hpt. However, when $k_5 \neq 0$ then sigmoidality can arise even if $k_{h1} = k_{3r} = 0$ but $k_{4r} > k_4$. For this model, necessary conditions for sigmoidality of the signal-response curve are either that

$$k_{h1} > 0 \quad \text{and} \quad k_2 > k_{2r} \quad \text{and} \quad (k_{3r} > k_3 \quad \text{or} \quad k_{3r}k_{4r} > k_3 k_4).$$

or that

$$k_5 > 0 \quad \text{and} \quad k_{4r} > k_4.$$

In other words, necessary conditions for sigmoidality of the signal-response curve are

$$k_{h1}(k_2 - k_{2r})(k_{3r} - k_3) \neq 0 \quad \text{or} \quad k_{h1}(k_2 - k_{2r})(k_{3r}k_{4r} - k_3 k_4) \neq 0 \quad \text{or} \quad k_5(k_{4r} - k_4) \neq 0.$$

2 The kinase HK is bifunctional

We consider the case in which the kinase HK is bifunctional, that is, HK acts as a phosphatase for REC.

2.1 Reactions, equations and steady states

Reactions. The minimal set of reactions that the system has, consists of the autophosphorylation reaction on HK and the forward phosphotransfer reactions

$$\mathrm{HK} \xrightarrow{k_s} \mathrm{HKp} \qquad \mathrm{HKp} + \mathrm{REC} \xrightarrow{k_2} \mathrm{HK} + \mathrm{RECp}$$

$$\mathrm{RECp} + \mathrm{Hpt} \xrightarrow{k_3} \mathrm{REC} + \mathrm{Hptp} \qquad \mathrm{Hptp} + \mathrm{RR} \xrightarrow{k_4} \mathrm{Hpt} + \mathrm{RRp},$$

together with the reaction for phosphatase activity of the histidine kinase HK:

$$\mathrm{HK} + \mathrm{RECp} \underset{k_{5r}}{\overset{k_5}{\rightleftarrows}} \mathrm{HKRECp} \xrightarrow{k_6} \mathrm{HK} + \mathrm{REC}.$$

We study the extensions of this minimal reaction mechanism obtained by adding reverse phosphotransfer reactions involving the HK, REC, Hpt and the RR, as well as hydrolysis reactions at RECp and RRp:

$$\mathrm{HK} + \mathrm{RECp} \xrightarrow{k_{2r}} \mathrm{HKp} + \mathrm{REC} \qquad \mathrm{RECp} \xrightarrow{k_{h1}} \mathrm{REC}$$

$$\mathrm{REC} + \mathrm{Hptp} \xrightarrow{k_{3r}} \mathrm{RECp} + \mathrm{Hpt} \qquad \mathrm{RRp} \xrightarrow{k_{h2}} \mathrm{RR}$$

$$\mathrm{Hpt} + \mathrm{RRp} \xrightarrow{k_{4r}} \mathrm{Hptp} + \mathrm{RR}.$$

By setting some of the rate constants k_{*r} or kh_* to zero, we obtain a total of 32 different topologies involving different combinations of reverse phosphorelay and hydrolysis reactions (Table 1 and Table S1), similarly to the situation where the HK kinase is monofunctional. The minimal set of reactions is always part of the system, meaning that $k_2, k_3, k_4, k_s \neq 0$ for all topologies.

Ordinary differential equations. We model the protein concentrations in the system using ordinary differential equations (ODEs). To simplify the notation, we define:

$$\begin{array}{lllll} x_1 = [\mathrm{HK}] & x_2 = [\mathrm{HKp}] & x_3 = [\mathrm{REC}] & x_4 = [\mathrm{RECp}] & \\ x_5 = [\mathrm{Hpt}] & x_6 = [\mathrm{Hptp}] & x_7 = [\mathrm{RR}] & x_8 = [\mathrm{RRp}] & x_9 = [\mathrm{HKRECp}]. \end{array}$$

The dynamics of the concentrations in time is modeled with a system of ODEs:

$$\begin{aligned}
\dot{x}_1 &= -k_s x_1 - k_{2r} x_1 x_4 + k_2 x_2 x_3 - k_5 x_1 x_4 + k_{5r} x_9 + k_6 x_9 \\
\dot{x}_2 &= k_s x_1 + k_{2r} x_1 x_4 - k_2 x_2 x_3 \\
\dot{x}_3 &= -k_2 x_2 x_3 + kh_1 x_4 + k_{2r} x_1 x_4 + k_3 x_4 x_5 - k_{3r} x_3 x_6 + k_6 x_9 \\
\dot{x}_4 &= k_2 x_2 x_3 - k_{h1} x_4 - k_{2r} x_1 x_4 - k_3 x_4 x_5 + k_{3r} x_3 x_6 - k_5 x_1 x_4 + k_{5r} x_9 \\
\dot{x}_5 &= -k_3 x_4 x_5 + k_{3r} x_3 x_6 + k_4 x_6 x_7 - k_{4r} x_5 x_8 \\
\dot{x}_6 &= k_3 x_4 x_5 - k_{3r} x_3 x_6 - k_4 x_6 x_7 + k_{4r} x_5 x_8 \\
\dot{x}_7 &= -k_4 x_6 x_7 + k_{h2} x_8 + k_{4r} x_5 x_8 \\
\dot{x}_8 &= k_4 x_6 x_7 - k_{h2} x_8 - k_{4r} x_5 x_8 \\
\dot{x}_9 &= k_5 x_1 x_4 - k_{5r} x_9 - k_6 x_9.
\end{aligned}$$

This system has four conserved amounts:

$$\overline{H} = x_1 + x_2 + x_9, \quad \overline{C} = x_3 + x_4 + x_9, \quad \overline{T} = x_5 + x_6, \quad \overline{R} = x_7 + x_8.$$

It is assumed that total amounts are positive.

Steady-state equations. We proceed as in the monofunctional case and conclude that the steady states of the system are given as the solutions to the following system of equations:

$$\overline{H} = x_1 + x_2 + x_9 \quad \text{(S28)}$$
$$\overline{C} = x_3 + x_4 + x_9 \quad \text{(S29)}$$
$$\overline{T} = x_5 + x_6 \quad \text{(S30)}$$
$$\overline{R} = x_7 + x_8 \quad \text{(S31)}$$

$$0 = k_s x_1 + k_{2r} x_1 x_4 - k_2 x_2 x_3 \quad \text{(S32)}$$
$$0 = k_2 x_2 x_3 - k_{h1} x_4 - k_{2r} x_1 x_4 - k_3 x_4 x_5 + k_{3r} x_3 x_6 - k_5 x_1 x_4 + k_{5r} x_9 \quad \text{(S33)}$$
$$0 = k_3 x_4 x_5 - k_{3r} x_3 x_6 - k_4 x_6 x_7 + k_{4r} x_5 x_8 \quad \text{(S34)}$$
$$0 = k_4 x_6 x_7 - k_{h2} x_8 - k_{4r} x_5 x_8 \quad \text{(S35)}$$
$$0 = k_5 x_1 x_4 - k_{5r} x_9 - k_6 x_9. \quad \text{(S36)}$$

Rearrangement of the steady-state equations. We change equations (S32)-(S36) by linear combinations of them. Specifically, we replace:

- (S34) by (S34)+(S35),
- (S33) by (S33)+(S34)+(S35)+(S36),
- (S32) by (S32)+(S33)+(S34)+(S35)+(S36),

and leave (S35) and (S36) as they are. This results in the following equivalent system of equations:

$$\overline{H} = x_1 + x_2 + x_9 \quad \text{(S37)}$$
$$\overline{C} = x_3 + x_4 + x_9 \quad \text{(S38)}$$
$$\overline{T} = x_5 + x_6 \quad \text{(S39)}$$
$$\overline{R} = x_7 + x_8 \quad \text{(S40)}$$

$$0 = k_s x_1 - k_{h1} x_4 - k_{h2} x_8 - k_6 x_9 \quad \text{(S41)}$$
$$0 = k_2 x_2 x_3 - k_{h1} x_4 - k_{2r} x_1 x_4 - k_{h2} x_8 - k_6 x_9 \quad \text{(S42)}$$
$$0 = k_3 x_4 x_5 - k_{3r} x_3 x_6 - k_{h2} x_8 \quad \text{(S43)}$$
$$0 = k_4 x_6 x_7 - k_{h2} x_8 - k_{4r} x_5 x_8 \quad \text{(S44)}$$
$$0 = k_5 x_1 x_4 - k_{5r} x_9 - k_6 x_9. \quad \text{(S45)}$$

Zero concentrations. We assume that $k_5, k_{5r}, k_6 \neq 0$, that is, the kinase is bifunctional and acts as a phosphatase for the dephosphorylation of the receiver protein. Additionally, we are assuming that $k_s, k_2, k_3, k_4 \neq 0$ and that all total amounts are positive.

In this scenario, for the topologies **topologies 9-13, 15**, the signal-response curve is constant and equals $x_8 = \overline{R}$. All the other topologies have non-constant signal-response curves and all concentrations at steady state are non-zero (see Appendix B.1).

The **topologies 17-24** ($k_{h1} = k_{h2} = 0$) exhibit signal-response curves defined piecewise and are treated differently. Zero steady-state values occur (see Appendix B.3).

2.2 Steady-state relations and signal-response curve

We proceed to find an expression for the signal-response curve. As in the previous case, the *signal* is taken to be the value of the rate constant k_s, and the *response* is the steady-state value of phosphorylated response regulator (x_8) corresponding to k_s (with all the other rate constants and total amounts fixed).

Contrary to the previous system, this case does not allow for an explicit analytical relation. Instead, we infer the existence of an analytical function relating k_s and x_8 and derive properties of this function. We start by establishing relations between each concentration x_i and x_8 at steady state.

Steady-state relations. We study here the steady-state solutions that do not have vanishing concentrations. We let

$$k_y = \frac{k_5}{k_{5r} + k_6} \quad \text{(S46)}$$

be the reverse of the Michaelis-Menten constant of HK. We express the concentrations $x_1, \dots, x_7$ at steady state in terms of x_8 and x_9, independently of k_s. In addition, we find a relation between x_8 and x_9 at steady state.

Expression	Behavior as function of x_8 and x_9
$x_7 = \overline{R} - x_8$	x_7 decreases in x_8
$x_6 = \frac{x_8(k_{4r}\overline{T}+k_{h2})}{k_4 x_7 + k_{4r} x_8}$	x_6 increases in x_8
$x_5 = \frac{k_4\overline{T}x_7 - k_{h2}x_8}{k_4 x_7 + k_{4r} x_8}$	x_5 decreases in x_8
$x_4 = \frac{k_{h2}x_8 + k_{3r}x_6(C-x_9)}{k_3 x_5 + k_{3r} x_6}$	x_4 increases in x_8 and decreases in x_9
$x_3 = \frac{k_3 x_5(C-x_9) - k_{h2}x_8}{k_3 x_5 + k_{3r} x_6}$	x_3 decreases in x_8 and in x_9
$x_2 = \frac{k_{2r}x_4(\overline{H}-x_9) + k_6 x_9 + k_{h1}x_4 + k_{h2}x_8}{k_2 x_3 + k_{2r} x_4}$	x_2 increases in x_8
$x_1 = \frac{k_2 x_3(\overline{H}-x_9) - k_6 x_9 - k_{h1}x_4 - k_{h2}x_8}{k_2 x_3 + k_{2r} x_4}$	x_1 decreases in x_8 and in x_9
$x_9 = g(x_8)$	

(S47)

The first seven rows of (S47) give an iterative way to find the steady-state values of concentrations $x_1, \dots, x_7$ once the values of x_8 and x_9 are known. The last entry gives the relation between x_9 and x_8. See Appendix B.2 for a proof.

The steady-state values are all positive if and only if x_8 is in the interval $(0, \alpha)$, where α is the first positive root of the degree-2 polynomial q_2 given in (S19) (that is, the same as for the monofunctional case).

In (S47), the variables $x_1, \dots, x_7$ are expressed as functions of x_8, x_9. The variable x_9 cannot explicitly be written as a function of x_8. The function g is known to exist, but we do not have an analytical expression of it. However, there is a procedure to obtain the steady-state value of x_9 corresponding to a given value of x_8. For each fixed x_8 strictly between 0 and α, x_9 is the first positive root of the following polynomial $G(x_8, x_9)$:

$$G(x_8, x_9) = c_0(x_8) + c_1(x_8)x_9 + c_2(x_8)x_9^2 + c_3(x_8)x_9^3,$$

where if we denote

$$z_1 := k_{3r}(k_{4r}T + k_{h2}), \quad z_2 := k_2 k_3(k_4 T + k_{h2}), \quad z_3 := k_{4r} - k_4, \quad z_4 := z_1 - k_3(k_4 T + k_{h2}),$$

then the coefficients $c_i(x_8)$ are:

$$\begin{aligned}
c_0(x_8) =& k_y x_8 (k_{h2}(k_4\overline{R} + z_3 x_8) + z_1\overline{C})\Big(k_{h2}(z_3(k_{h1} + k_2\overline{H}) + z_4)x_8^2 \\
&+ (\overline{C}(k_{h1}z_1 + z_2\overline{H}) + k_{h2}k_4\overline{R}(k_2\overline{H} + k_3\overline{T} + k_{h1}))x_8 - k_2k_3k_4\overline{CHTR}\Big) \\
c_1(x_8) =& \Big((k_{2r}z_1 - z_2)\overline{C}x_8 - k_{h2}(k_2 - k_{2r})(k_4\overline{R} + z_3x_8)x_8 + k_2k_3k_4\overline{CTR}\Big)(k_3k_4\overline{TR} + z_4x_8) \\
&+ \Big((k_2k_3k_4\overline{TR} - x_8z_2)(k_{h2}(x_8z_3 + k_4\overline{R})(\overline{H} + \overline{C}) + z_1(2\overline{H} + \overline{C})\overline{C}) \\
&- k_{h2}k_2z_1(x_8z_3 + k_4\overline{R})(\overline{H} + \overline{C})x_8 - k_2k_{h2}^2x_8(x_8^2z_3^2 + k_4^2\overline{R}^2) \\
&- 2k_{h2}z_3(k_2k_4k_{h2}\overline{R} + z_1k_{h1})x_8^2 + k_{h2}x_8(k_6z_3 - z_1)(k_3k_4\overline{TR} + z_4x_8) \\
&+ (k_6z_4x_8 - 2z_1k_{h1}x_8 + k_3k_4k_6\overline{TR})(z_1\overline{C} + k_{h2}k_4\overline{R})\Big)x_8k_y \\
c_2(x_8) =& \Big(k_{h2}z_3(z_2 + k_2z_1)x_8^2 + (k_{h1}z_1^2 + (2z_2\overline{C} + k_2k_{h2}k_4\overline{R} - k_6z_4 + z_2\overline{H})z_1 \\
&- k_4k_{h2}\overline{R}(k_2k_3z_3\overline{T} - z_2))x_8 - k_3k_4\overline{RT}(k_2k_4k_{h2}\overline{R} + z_1(k_6 + 2\overline{C}k_2 + k_2\overline{H}))\Big)x_8k_y \\
&- (k_3k_4\overline{TR} + z_4x_8)(k_2k_3k_4\overline{TR} + (k_{2r}z_1 - z_2)x_8) \\
c_3(x_8) =& z_1(k_2k_3k_4\overline{TR} - z_2x_8)k_yx_8
\end{aligned}$$

Signal-response expression. Using the remaining steady-state equation, (S41), we express k_s in terms of x_8:

$$k_s = f_b(x_8) = \frac{k_{h1}x_4 + k_{h2}x_8 + k_6x_9}{x_1}, \tag{S48}$$

where x_1, x_4, x_9 are given in terms of x_8 as well (see (S47) above) and $x_1 \neq 0$ at steady state. If x_8 approaches α, then x_1 tends to zero and k_s tends to infinity. It follows that α is precisely the maximal response of x_8.

The function f_b is continuous and differentiable in $[0, \alpha)$ and is strictly increasing (see Appendix B.3). It admits an inverse

$$\varphi_b = f_b^{-1},$$

which is the signal-response curve. The signal-response curve is increasing, continuous and differentiable in $[0, +\infty)$. When k_s tends to infinity then the response x_8 tends to α.

If $k_{h1} = k_{h2} = 0$ then φ_b is defined by f_b^{-1} if $k_s \in [0, k_6k_y\overline{C}]$ and $\varphi_b = \overline{R}$ for $k_s > k_6k_y\overline{C}$.

Practical considerations. In order to plot the signal-response curve we use the following procedure:

(i) Compute α (the first positive root of $q_2(x_8)$ in x_8) and choose a grid of values for x_8, strictly between 0 and α.

(ii) For each value of x_8, find the first positive root of $G(x_8, x_9)$ as a function of x_9, that is, for each value of x_8 we find a value of x_9.

(iii) Compute x_1, x_4 using (S47) and the pair of values (x_8, x_9).

(iv) Compute k_s using (S48) in terms of x_1, x_4, x_8, x_9.

In this way, points on the signal-response curve $(f_b(x_8), x_8)$ are generated. Because of the relationship between f_b and φ_b, the points give a plot of the function φ_b.

Comparison of the monofunctional and bifunctional cases. We have shown that the maximal response of phosphorylated response regulator $x_8 = \mathrm{RRp}$ is independent of whether the kinase is bifunctional or not. In particular, the value is independent of the rate constants k_5, k_{5r}, k_6.

However, the signal-response curve in the bifunctional case is always below the signal-response curve in the monofunctional case. Indeed, if the kinase is monofunctional, then the expressions in (S18) can be obtained from those in (S47) by setting $x_9 = 0$. It follows that if the common reactions have the same rate constants in the two cases, then for every fixed x_8, the value of x_4 (resp. x_1) in the bifunctional case is smaller (resp. larger) than in the monofunctional case. Consequently, for any rate constants k_5, k_{5r}, k_6, we have $f_b(x_8) < f(x_8)$. In other words, the signal k_s required to achieve a certain response x_8 is smaller in the monofunctional case than in the bifunctional case. Nevertheless, as k_s increases, the steady-state value of x_8 tends to the maximal response (which is the same value in both cases). Therefore, the difference between the steady-state value of x_8 in the two cases becomes negligible for large values of k_s.

This is due to sequestration of substrate in x_9. Therefore, the signal-response curve in the monofunctional case is always above the corresponding curve in the bifunctional case (for any choice of additional rate parameters). Since the maximal response is independent of the role of the kinase, a smaller signal is required to get close to the maximal response, when the kinase is monofunctional. Furthermore, if the inverse of the Michaelis-Menten constant $k_y = k_5/(k_{5r} + k_6)$ (equation (S46)) increases and k_6 is fixed, then $f_b(x_8)$ increases for a fixed x_8 (see Appendix B.3).

2.3 Hyperbolic and sigmoidal signal-response curves

We apply the same indicator to classify a curve as sigmoidal or hyperbolic as in the previous case. That is, we calculate the sign of the second derivative of the signal-response curve at zero and classify the curve accordingly. We have computed $\varphi_b''(0)$ using the method introduced in Subsection 1.5.

If $\varphi_b''(0) > 0$ then we classify the signal-response curve as sigmoidal, and if $\varphi_b''(0) < 0$ then we classify the signal-response curve as sigmoidal.

The sign of the second derivative of φ_b at 0 agrees with the sign of

$$S_b = S + H(\alpha_2 H + \alpha_3)k_y + \alpha_1 H k_y^2 \tag{S49}$$

where $k_y = \frac{k_5}{k_{5r}+k_6}$ and

$$\begin{aligned}
\alpha_1 =& C(k_{4r}T + k_{h2})H^2 k_2 k_6 k_{3r}(k_{3r}C(k_{4r}T + k_{h2}) + k_4 k_{h2} R)\\
\alpha_2 =& -C(k_{4r}T + k_{h2})k_2(Ck_3k_6k_{3r}(k_4T + k_{h2}) - k_{3r}^2(k_{h1}C + k_6)(k_{4r}T + k_{h2})\\
&+ k_4k_{h2}R(k_6(k_3 - k_{3r}) - k_{h1}k_{3r}))\\
\alpha_3 =& -(k_{3r}C(k_{4r}T + k_{h2}) + k_4k_{h2}R)(k_{3r}k_{h1}(k_{4r}CT + k_{h2}) + k_4k_{h2}R(k_3T + k_{h1}))(k_2C + k_6)
\end{aligned}$$

The term α_1 is always positive and the term α_3 is always negative. The independent term (obtained by setting $k_y = 0$) is identical to the term given in the monofunctional case (S22). We have that if $k_{3r} = 0$, then $S_b < 0$ and the function φ_b is hyperbolic. However, when $k_{h1} = 0$ the system can show sigmoidality (because $\alpha_1 \neq 0$).

Observe that the leading coefficient of the term S_b in (S49) seen as a polynomial of degree 2 in k_y is positive. Therefore, by increasing k_y enough, S_b becomes positive and the curve sigmoidal. Recall that k_y is the inverse of the Michaelis-Menten constant of the enzyme HK for its dephosphorylation

activity. Therefore, increasing k_y corresponds to making the enzyme mediated dephosphorylation of RECp more efficient.

A Proof of the claims: monofunctional case

This appendix provides the proofs of the claims in the Section 1.

A.1 Constant signal-response curves and zero solutions

- $k_{h1} = k_{h2} = 0$: From (S14) we have that $x_1 = 0$ (since $k_s \neq 0$) and thus $x_2 = \overline{H}$ at steady state. From (S15) it follows that $x_3 = 0$ (since $k_2 \neq 0$) and thus $x_4 = \overline{C}$. From (S16) we have that $x_5 = 0$ (since $k_3 \neq 0$) and so $x_6 = \overline{T}$ and finally from (S17) it follows that $x_7 = 0$ (since $k_4 \neq 0$) and $x_8 = \overline{R}$. This fact is independent of the value of all other constants.

- $k_{h2} = k_{3r} = 0$: From (S16) either $x_4 = 0$ or $x_5 = 0$. If $x_4 = 0$ we have that $x_3 \neq 0$. From (S14), $x_1 = 0$ and from (S15) we have $x_2 = 0$ which is a contradiction. Therefore $x_5 = 0$ and consequently, $x_6 = \overline{T} \neq 0$ and $x_7 = 0$. Thus $x_8 = \overline{R}$ and $x_1, \dots, x_4$ fulfill (S14), (S15) and the equations for the total amounts. Additionally, the signal-response curve for Hpt is also constant (that is, at steady state $x_6 = \overline{T}$), but the curves corresponding to HK and REC are not constant.

- $k_{h2} = k_{4r} = 0$: From (S17) either $x_6 = 0$ or $x_7 = 0$. If $x_6 = 0$ we have that $x_5 \neq 0$. From (S16) $x_4 = 0$ and from (S14) we have $x_1 = 0$. By (S15) we have $x_2 x_3 = 0$ which is a contradiction. Therefore $x_7 = 0$, and then $x_8 = \overline{R} \neq 0$. $x_1, \dots, x_6$ fulfill (S14)-(S16) and the equations for the total amounts. In this case the signal-response curves for HK, REC and Hpt are not constant.

Assume that none of the cases above hold, that is, that either $k_{h2} \neq 0$ or $k_{h2} = 0$ and $k_{h1} k_{3r} k_{4r} \neq 0$. Assume that all total amounts are positive. We show that in this case any non-negative solution to the steady-state equations is positive, that is, all concentrations are non-zero. As a consequence, the signal-response curve cannot be constant (equal to $\overline{R}$; because this would imply $x_7 = 0$).

- If $x_1 = 0$, then by (S14) it must be that $k_{h1} x_4 = k_{h2} x_8 = 0$ and $x_2 \neq 0$. From (S15) it follows that $x_3 = 0$ and hence $x_4 \neq 0$ (because $\overline{C} > 0$) and thus $k_{h1} = 0$. In this case $k_{h2} \neq 0$ and hence $x_8 = 0$. From (S16) we have $x_5 = 0$ and hence $x_6 \neq 0$. From (S17) we see that $x_7 = 0$ contradicting $\overline{R} > 0$.

- If $x_2 = 0$ or $x_3 = 0$ then from (S15) we have $k_{h1} x_4 = k_{h2} x_8 = k_{2r} x_1 x_4 = 0$. It follows from (S14) that $x_1 = 0$ and we reach a contradiction with the item above.

- If $x_4 = 0$ or $x_5 = 0$ then from (S16) we have $k_{3r} x_3 x_6 = k_{h2} x_8 = 0$. If $x_4 = 0$ using (S14) we have that $k_s x_1 = 0$ and hence $x_1 = 0$, which is a contradiction. If $x_5 = 0$ then from (S17) we have $k_4 x_6 x_7 = 0$. Since $x_6 \neq 0$ (because $\overline{T} > 0$) and $k_4 \neq 0$ by hypothesis, we have $x_7 = 0$. As a consequence $x_8 \neq 0$. Hence $k_{h2} = 0$ and by hypothesis $k_{h1} k_{3r} k_{4r} \neq 0$. If $k_{3r} \neq 0$ we have $x_3 = 0$ contradicting the item above.

- If $x_6 = 0$ or $x_7 = 0$ then $k_{h2} x_8 = k_{4r} x_5 x_8 = 0$. Since we showed that $x_5 \neq 0$, and $k_{h2} k_{4r} \neq 0$ by hypothesis, it follows that $x_8 = 0$. If $x_7 = 0$ we reach a contradiction. If $x_6 = 0$ then using (S16) we have $x_4 x_5 = 0$ which contradicts the item above.

- If $x_8 = 0$ then $x_7 \neq 0$ and hence by (S17) $x_6 = 0$ which contradicts the item above.

A.2 Steady-state relations

Here we derive the relations shown in (S18).

(1) From the total amount $\overline{R}$, we have

$$x_7 = \overline{R} - x_8. \tag{S50}$$

This expression shows that x_7 decreases in x_8. For $x_7, x_8 > 0$ we require $0 < x_8 < \overline{R}$. Hence, if we let $\alpha_1 = \overline{R}$ and $I_1 = (0, \alpha_1)$, any positive steady state must satisfy $x_8 \in I_1$.

(2) Solving (S17) for x_6 and subsequently solving x_5 using the total amount $\overline{T}$, we obtain:

$$x_6 = \frac{(k_{h2} + k_{4r}\overline{T})x_8}{k_4 x_7 + k_{4r} x_8}, \qquad x_5 = \frac{k_4\overline{T}x_7 - k_{h2}x_8}{k_4 x_7 + k_{4r} x_8}. \tag{S51}$$

The expression of x_5 decreases in x_8 and increases in x_7. Since x_7 decreases in x_8, we conclude that after substituting x_7 with (S50), x_5 decreases in x_8. Similarly, x_6 increases in x_8.

Assume that $k_{h2} \neq 0$. For $x_5 > 0$ we require $k_4\overline{T}x_7 > k_{h2}x_8$. When $x_8 = 0$ this inequality holds. The right-hand side of the inequality increases in x_8 and goes to infinity. The left-hand side decreases in x_8 and is zero when $x_8 = \alpha_1$. Therefore, there exists a unique value of x_8, $\alpha_2 < \alpha_1$ at which $k_4\overline{T}x_7 = k_{h2}x_8$. Then, the inequality holds if and only if $x_8 < \alpha_2$. If $k_{h2} = 0$, then $x_5 > 0$ for all $x_8 \in I_1$ and we define $\alpha_2 = \alpha_1$.

Let $I_2 = (0, \alpha_2)$. Since $\alpha_2 \leq \alpha_1$, if $x_8 \in I_2$ then $x_5, \ldots, x_8 > 0$. Observe that if $x_8 = \alpha_2$, then $x_5 = 0$. If $x_8 = 0$, then $x_5 = \overline{T}$.

(3) Using (S16) to express x_4 in terms of x_5, x_6 and x_8 and using the total amount $\overline{C}$ we obtain

$$x_4 = \frac{k_{3r}\overline{C}x_6 + k_{h2}x_8}{k_3 x_5 + k_{3r} x_6}, \qquad x_3 = \frac{k_3\overline{C}x_5 - k_{h2}x_8}{k_3 x_5 + k_{3r} x_6}. \tag{S52}$$

The expression of x_3 decreases in x_6, x_8 and increases in x_5. Since x_5 decreases in x_8, and x_6 increases in x_8, we conclude that after substituting x_5, x_6 with (S51), x_3 decreases in x_8. Similarly, x_4 increases in x_8.

We proceed to discuss positivity following the reasoning above. Assume that $k_{h2} \neq 0$. Then for $x_3 > 0$ we require

$$k_3\overline{C}x_5 > k_{h2}x_8.$$

The inequality holds at $x_8 = 0$. The right-hand side of the inequality increases in x_8 and goes to infinity. The left-hand side decreases in x_8 and is zero when $x_8 = \alpha_2$. Therefore, there exists a unique value of x_8, $\alpha_3 < \alpha_2$ at which $k_3\overline{C}x_5 = k_{h2}x_8$ and the inequality holds if and only if $x_8 < \alpha_3$. If $k_{h2} = 0$, then $x_5 > 0$ for all $x_8 \in I_2$ and $\alpha_3 = \alpha_2$.

Let $I_3 = (0, \alpha_3)$. Since $\alpha_3 \leq \alpha_2$, we have $x_3, \ldots, x_8 > 0$ if and only if $x_8 \in I_3$. Note that if $x_8 = \alpha_3$, then $x_3 = 0$. If $x_8 = 0$ then $x_3 = \overline{C}$.

(4) Using (S15) and the total amount $\overline{H}$ and obtain:

$$x_2 = \frac{k_{2r}\overline{H}x_4 + k_{h1}x_4 + k_{h2}x_8}{k_2 x_3 + k_{2r} x_4}, \qquad x_1 = \frac{k_2\overline{H}x_3 - k_{h1}x_4 - k_{h2}x_8}{k_2 x_3 + k_{2r} x_4} \tag{S53}$$

The expression of x_1 decreases in x_4, x_8 and increases in x_3. Since x_3 decreases in x_8, and x_4 increases in x_8, we conclude that after substituting x_3, x_4 with (S52), x_1 decreases in x_8. Similarly, x_2 increases in x_8.

For $x_1 > 0$ we require

$$k_2\overline{H}x_3 > k_{h1}x_4 + k_{h2}x_8.$$

Recall that by hypothesis we have either $k_{h1} \neq 0$ or $k_{h2} \neq 0$, that is, the two hydrolysis rate constants cannot vanish simultaneously. If $x_8 = 0$ then the inequality is satisfied (because $x_4 = 0$). The right-hand side of the inequality increases in x_8 and goes to infinity. The left-hand side decreases in x_8 and is zero when $x_8 = \alpha_3$. Therefore, there exists a unique value of x_8 in I_3, $\alpha < \alpha_3$, at which $k_2\overline{H}x_3 = k_{h1}x_4 + k_{h2}x_8$ and the inequality holds if and only if $x_8 < \alpha$.

Let $I = (0, \alpha)$. Since $\alpha \leq \alpha_3$, we have $x_1, \dots, x_8 > 0$ if and only if $x_8 \in I$. Note that if we let $x_8 = \alpha$, then $x_1 = 0$ and if $x_8 = 0$ then $x_1 = \overline{H}$.

The value α is the first positive value of x_8 for which

$$k_2\overline{H}x_3 - k_{h1}x_4 - k_{h2}x_8 = 0. \tag{S54}$$

After substituting x_3, x_4 by (S52), and subsequently by (S51) and (S50), the left-hand side of this equality is a quotient of polynomials. The first positive root (in x_8) of the numerator is α. The numerator is precisely the polynomial $q_2(x_8)$ given in (S19).

A.3 Signal-response curve

The entries of (S18) are derived using all steady-state equations except for (S14). From (S14) we obtain that

$$k_s = \frac{k_{h1}x_4 + k_{h2}x_8}{x_1}. \tag{S55}$$

The expression of k_s is positive provided that $x_8 \in I$. Since x_4 increases in x_8 and x_1 decreases in x_8, we see that k_s is expressed as an increasing positive function for $x_8 \in I$. When x_8 approaches the upper bound of the interval I, α, then x_1 tends to zero and x_4 to some finite number. Hence k_s grows to infinity.

The explicit form of f given in (S20) is obtained from (S55) by plugging in the values of $x_1, \dots, x_7$ obtained in (S50)-(S53) (computations are done in Mathematica).

A.4 Hyperbolic shape when phosphorelay rates are large

We prove here that if (S24) holds, then the second derivative of φ at any value of k_s is negative.

Using Mathematica, we compute the second derivative of x_4 with respect to x_8 and find that its sign equals the sign of

$$(k_3 - k_{3r})(k_4k_{h2}\overline{R} + k_{3r}k_{h2}\overline{C}) + k_{3r}(k_3k_4 - k_{3r}k_{4r})\overline{CT}.$$

Consider now

$$x_2 = \frac{k_{2r}\overline{H}x_4 + k_{h1}x_4 + k_{h2}x_8}{k_2x_3 + k_{2r}x_4} = \frac{(k_{2r}\overline{H} + k_{h1})x_4 + k_{h2}x_8}{(k_{2r} - k_2)x_4 + k_2\overline{C}}.$$

Let $\beta = (k_{2r}\overline{H} + k_{h1})x_4 + k_{h2}x_8$ be the numerator of x_2 and $\gamma = (k_{2r} - k_2)x_4 + k_2\overline{C}$ be the denominator of x_2. Both terms are positive. We take the second derivative of x_2 with respect to x_8 and obtain:

$$\begin{aligned} x_2'' &= \frac{(\beta''\gamma - \beta\gamma'')\gamma - 2\gamma'(\beta'\gamma - \beta\gamma')}{\gamma^3} \\ &= \frac{((k_{2r}\overline{H} + k_{h1})\gamma - (k_{2r} - k_2)\beta)\gamma x_4'' - 2(k_{2r} - k_2)x_4'(\beta'\gamma - \beta\gamma')}{\gamma^3}. \end{aligned}$$

Let

$$A = ((k_{2r}\overline{H} + k_{h1})\gamma - (k_{2r} - k_2)\beta)\gamma x_4'' = (k_{2r}(\overline{H}\gamma - \beta) + k_{h1}\gamma + k_2\beta)\gamma x_4'',$$
$$B = -2(k_{2r} - k_2)x_4'(\beta'\gamma - \beta\gamma')$$

such that $x_2'' = (A+B)/\gamma^3$. The denominator is positive. Therefore, the sign of x_2'' is determined by the sign of $A+B$. Since x_2, x_4 increase in x_8 we have that $\beta'\gamma - \beta\gamma' > 0$ and $x_4' > 0$. Therefore, the sign of B equals the sign of $k_2 - k_{2r}$.

The term $\overline{H}\gamma - \beta = -k_2 x_4 \overline{H} + k_2 \overline{CH} - k_{h1}x_4 - k_{h2}x_8$ is positive in I because it agrees with the numerator of x_1. It follows that the sign of A equals the sign of x_4''. If the signs of A and B agree, then x_2'' has a constant sign over I.

Consider now the inverse of the signal-response curve:

$$k_s = f(x_8) := \frac{k_{h1}x_4 + k_{h2}x_8}{x_1}$$

and let $\delta = k_{h1}x_4 + k_{h2}x_8$. The second derivative of f with respect to x_8 is

$$f'' = \frac{x_1(k_{h1}x_4''x_1 - \delta x_1'') \; - 2x_1'(\delta' x_1 - \delta x_1')}{x_1^3}.$$

The term $-2x_1'(\delta' x_1 - \delta x_1')$ is positive because $x_1' < 0$ and $(\delta' x_1 - \delta x_1') > 0$ (it is the numerator of the derivative of f). If $(k_{h1}x_4''x_1 - \delta x_1'') > 0$, then the signal-response curve is hyperbolic (because the sign of the second derivative of φ is minus the sign of the second derivative of f). In particular, this is the case if $x_4'' > 0$ and $x_1'' < 0$. For $x_1'' < 0$ we require $x_2'' > 0$.

Therefore, if $x_2'', x_4'' > 0$, then the signal-response curve is hyperbolic. Using the computations above, we conclude that if

$$k_2 - k_{2r}, \qquad \text{and} \qquad (k_3 - k_{3r})(k_4 k_{h2}\overline{R} + k_{3r}k_{h2}\overline{C}) + k_{3r}(k_3k_4 - k_{3r}k_{4r})\overline{CT} > 0,$$

then the curve is hyperbolic. These two inequalities are in particular fulfilled if

$$k_2 > k_{2r}, \qquad k_3 > k_{3r}, \qquad k_4 > k_{4r},$$

that is, if the forward phosphorelay rate constants are larger than their reverse counterparts.

B Proof of the claims: bifunctional case

This appendix provides a sketch of the proofs of the claims in Section 2.

B.1 Zero concentrations

We start by checking that the combinations $k_{h2} = 0$ and either $k_{3r} = 0$ or $k_{4r} = 0$ provide constant signal-response curves. Assume that $k_{h2} = 0$ and $x_4 = 0$ at steady state. Then by (S45) $x_9 = 0$. Consequently from (S41) we have $x_1 = 0$ and hence $x_2 \neq 0$ (S37). Similarly from (S38) we have that $x_3 \neq 0$. But then (S42) cannot hold. Therefore, if $k_{h2} = 0$, $x_4 \neq 0$ at steady state.

- $k_{h2} = k_{3r} = 0$: From (S43) $x_5 = 0$ because $x_4 \neq 0$ at steady state. Consequently, $x_6 = \overline{T} \neq 0$ and from (S44) it follows that $x_7 = 0$. Thus $x_8 = \overline{R} \neq 0$ at steady state.

- $k_{h2} = k_{4r} = 0$: From (S44) either $x_6 = 0$ or $x_7 = 0$. If $x_6 = 0$ we have that $x_5 \neq 0$. From (S43) if follows $x_4 = 0$, which is a contradiction. Therefore $x_7 = 0$, and hence $x_8 = \overline{R} \neq 0$ at steady state.

Assume now that none of the two scenarios above occur, and further that $k_{h1} = k_{h2} = 0$ does not occur. If $x_9 = 0$ is a solution at steady state, then by (S45) we must either have $x_1 = 0$ or $x_4 = 0$. If $x_1 = 0$ then $x_2 \neq 0$. Further from (S41) we have $k_{h1}x_4 = k_{h2}x_8 = 0$. Hence from (S42) we have $x_3 = 0$ and as a consequence $x_4 \neq 0$. If $k_{h1} \neq 0$ then $k_{h1}x_4 \neq 0$, which is a contradiction. Hence, assume that $k_{h1} = 0$. Then using (S43) we deduce that $x_5 = 0$ and hence $x_6 \neq 0$ from the conservation law. From (S44) we obtain $x_7 = 0$ which contradicts (S40) for $\overline{R} > 0$ only if $k_{h2} \neq 0$.

Assume now that $x_4 = 0$. Then by (S38) we have $x_3 \neq 0$. Further, from (S43) we have $k_{h2}x_8 = 0$. From (S42) we have $x_2 = 0$ and from (S37) $x_1 \neq 0$, contradicting (S41).

Therefore, if $k_{h1} \neq 0$ or $k_{h2} \neq 0$ then $x_9 = 0$ is not a solution at steady state. If one of the concentrations $x_1, \ldots, x_4$ is zero at steady state, then the positive term in one of the equations (S42),(S43),(S45) vanishes, implying that all the other monomials must vanish as well. For any of the equations, it would follow imply that $k_6x_9 = 0$ contradicting $x_9 \neq 0$. That is, $x_1, \ldots, x_4 \neq 0$ at steady state. If $x_5 = 0$ and $k_{3r} \neq 0$ then using (S43) and (S39), $x_3 = 0$ which is a contradiction. If $k_{3r} = 0$ then $k_{h2} \neq 0$ (by assumption) and hence $x_8 = 0$. By (S44) we have $x_6 = 0$ or $x_7 = 0$. The latter contradicts (S40) because $x_8 = 0$. Hence $x_6 = 0$. However this contradicts (S39), because $x_5 = 0$.

Therefore, if $k_{h2} \neq 0$ or if $k_{h2} = 0$ but $k_{3r}k_{4r}k_{h1} \neq 0$, then there are no zero concentrations at steady state.

B.2 Steady-state relations

Here we derive the expressions shown in (S47). We study the concentrations at steady state that are non-zero. We assume either (1) $k_{h2} \neq 0$ or (2) $k_{h2} = 0$ and $k_{3r}k_{4r}k_{h1} \neq 0$.

(1) Using the total amount equation for $\overline{R}$, we have

$$x_7 = \overline{R} - x_8, \tag{S56}$$

such that x_7 is expressed as a decreasing function of x_8. We have $x_7, x_8 > 0$ if and only if $0 < x_8 < \alpha_1 := \overline{R}$.

(2) Using (S44) and the total amount equation for $\overline{T}$ we obtain

$$x_6 = \frac{x_8(k_{4r}\overline{T} + k_{h2})}{k_4x_7 + k_{4r}x_8}, \qquad x_5 = \frac{k_4\overline{T}x_7 - k_{h2}x_8}{k_4x_7 + k_{4r}x_8}. \tag{S57}$$

The expression for x_5 decreases in x_8 and increases in x_7. Since x_7 decreases in x_8, we conclude that after substituting x_7 with (S56), x_5 decreases in x_8. Similarly x_6 increases in x_8.

For $x_5, x_6, x_7 > 0$, we require $k_4\overline{T}(\overline{R} - x_8) - k_{h2}x_8 > 0$, that is,

$$0 < x_8 < \alpha_2 := \frac{k_4\overline{T}\overline{R}}{k_4\overline{T} + k_{h2}} \leq \alpha_1.$$

Hence, $0 < x_8 < \alpha_2$ if and only if $x_5, x_6, x_7, x_8 > 0$.

(3) Using (S43) and the total amount equation for $\overline{C}$, we obtain:

$$x_4 = \frac{k_{h2}x_8 + k_{3r}x_6(C - x_9)}{k_3x_5 + k_{3r}x_6}, \qquad x_3 = \frac{k_3x_5(C - x_9) - k_{h2}x_8}{k_3x_5 + k_{3r}x_6}. \tag{S58}$$

x_4 is positive provided $0 < x_8 < \alpha_2$ and $0 < x_9 < C$. x_3 is positive provided x_8, x_9 satisfy $k_3x_5(C - x_9) > k_{h2}x_8$, that is,

$$x_9 < \frac{k_3x_5C - k_{h2}x_8}{k_3x_5} \leq C.$$

The right-hand side decreases in x_8. It is zero when $k_3x_5C = k_{h2}x_8$. If $x_8 > 0$ then $x_5 > 0$ and hence the value that makes the right-hand side zero satisfies $x_8 < \alpha_2$. Therefore, $x_3, \dots, x_9$ are positive for x_8, x_9 in the set

$$\Omega_1 := \left\{(x_8, x_9) \in \mathbb{R}^2_+ | x_9 < \frac{k_3x_5C - k_{h2}x_8}{k_3x_5}\right\},$$

with x_5 given by (S56) and (S57). For each value of $0 < x_9 < C$, let $\beta(x_9)$ be the value of x_8 for which $x_9 = \frac{k_3x_5C - k_{h2}x_8}{k_3x_5}$, that is, the upper-bound of allowed values for x_8. Note that $\beta(x_9)$ decreases in x_9.

The expression for x_4 in (S58) increases in x_8 and decreases in x_5, x_9. The derivative of x_4 with respect to x_6 equals

$$\frac{k_{3r}x_3}{(k_3x_5 + k_{3r}x_6)},$$

and hence it is positive provided $x_3, x_5, x_6 > 0$. Therefore, for $(x_8, x_9) \in \Omega_1$, x_4 increases in x_8 and decreases in x_9. Similarly, x_3 decreases in x_8 and in x_9.

(4) Using (S42) and the total amount equation for $\overline{H}$ we obtain

$$x_1 = \frac{k_2x_3(\overline{H} - x_9) - k_6x_9 - k_{h1}x_4 - k_{h2}x_8}{k_2x_3 + k_{2r}x_4}, \quad x_2 = \frac{k_{2r}x_4(\overline{H} - x_9) + k_6x_9 + k_{h1}x_4 + k_{h2}x_8}{k_2x_3 + k_{2r}x_4}.$$

For $(x_8, x_9) \in \Omega_1$, x_1 is positive provided that

$$k_2x_3(\overline{H} - x_9) > k_{h1}x_4 + k_{h2}x_8 + k_6x_9.$$

Fix a value of $0 < x_9 < \min(\overline{H}, \overline{C})$. Then the left-hand side of the inequality is a decreasing function of x_8 and the right-hand side of the inequality is increasing in x_8. It follows that there exists a value $\gamma(x_9)$ such that the inequality is fulfilled if and only if $x_8 < \gamma(x_9)$.

If $x_8 = \beta(x_9)$, then $x_3 = 0$ while the right-hand side of the inequality is positive. It follows that $\beta(x_9) > \gamma(x_9)$. x_2 is positive if x_4, x_3 are positive and $x_9 < H$. Therefore, $x_1, \dots, x_9$ are positive provided x_8, x_9 belong to

$$\Omega_2 := \{(x_8, x_9) \in \mathbb{R}^2_+ | \ x_9 < \min(\overline{H}, \overline{C}), \ k_2x_3(\overline{H} - x_9) > k_{h1}x_4 + k_{h2}x_8 + k_6x_9\}.$$

It can be seen that in Ω_2, x_1 decreases in x_9 and in x_8. Similarly, x_2 increases in x_8. Further, the numerator of x_1 also decreases in x_9. It follows that the supremum of x_8 in Ω_2 is obtained by setting $x_9 = 0$:

$$k_2x_3\overline{H} - k_{h1}x_4 - k_{h2}x_8 = 0,$$

where $x_9 = 0$ is inserted into the expression of x_3, x_4. The solution of this equation is precisely the value α obtained in the monofunctional case (see equation (S54)). Furthermore, the possible values of x_8 in Ω_2 are in $I = (0, \alpha)$.

(5) Using (S45) we obtain another expression for x_1 at steady state:

$$x_1 = \frac{(k_{5r} + k_6)x_9}{k_5 x_4} = \frac{x_9}{k_y x_4},$$

where $k_y = k_5/(k_{5r} + k_6)$. This expression decreases in x_8. We equate the two expressions for x_1:

$$\frac{x_9}{k_y x_4} = \frac{k_2 x_3 \overline{H} - k_2 x_3 x_9 - k_6 x_9 - k_{h1} x_4 - k_{h2} x_8}{k_2 x_3 + k_{2r} x_4} \quad \text{(S59)}$$

in order to relate x_8 and x_9. This equality does not provide a linear equation in x_8 nor in x_9, when substituting the expressions for x_3, x_4 in terms of x_8, x_9. Thus, we have to proceed in a different way from what we have done so far. For a fixed value of x_8 in I, the left-hand side of the equation increases in x_9 and the right-hand side decreases in x_9. Therefore, for a fixed value of x_8 in $I = (0, \alpha)$, the two sides of the equality intersect in exactly one point: $x_9 = g(x_8)$. Since the intersection point ensures that the right-hand side is positive, the intersection point satisfies by construction that $(x_8, g(x_8)) \in \Omega_2$.

We do not have an analytical description of g but we have a procedure to determine $g(x_8)$ from a given x_8. The function g is given by the Implicit Function Theorem. Let

$$G(x_8, x_9) = (k_2 x_3(\overline{H} - x_9) - k_6 x_9 - k_{h1} x_4 - k_{h2} x_8) k_y x_4 - x_9(k_2 x_3 + k_{2r} x_4) = 0.$$

Then, for every value of x_8, $g(x_8)$ is the first positive root of $G(x_8, x_9)$. It follows that g is continuous in I and differentiable. The derivative of g with respect to x_8 is given by

$$g'(x_8) = -\frac{(\partial G/\partial x_8)(x_8, g(x_8))}{(\partial G/\partial x_9)(x_8, g(x_8))}.$$

The function g can be extended to $x_8 = 0$ with $g(0) = 0$.

B.3 Signal-response curve

The entries of (S47) are derived using all steady-state equations except for (S41). From (S41) we obtain that

$$k_s = f_b(x_8) = \frac{k_{h1} x_4 + k_{h2} x_8 + k_6 x_9}{x_1}. \quad \text{(S60)}$$

If $x_8 \in I$, then $f_b(x_8)$ is positive. Therefore, all concentrations at steady state are positive. This function is continuous and differentiable. When x_8 approaches the upper bound of the interval I, α, then x_1 tends to zero, x_4 to some finite number and x_9 to zero. Hence k_s grows to infinity (provided k_{h1} or k_{h2} are non-zero, see below for the case $k_{h1} = k_{h2} = 0$). It follows that the image of f_b is $(0, +\infty)$ which guarantees the existence of at least one steady state. The function f_b can be differentiably extended at zero such that $f_b(0) = 0$.

Using the Chemical Reaction Network toolbox [2], we know that the system does not admit multiple positive steady states. By continuity, it follows that f_b must be monotone, that is, an increasing function. By the Inverse Function Theorem, there exists a continuous and differentiable function in $(0, +\infty)$,

$$x_8 = \varphi_b(k_s)$$

defined by $\varphi_b(k_s) = x_8$ if and only if $k_s = f_b(x_8)$.

If we increase k_y while keeping k_6 fixed, the right-hand side of (S59) increases. It follows that the value $g(x_8)$ increases. Consequently, x_4 increases and x_1 decreases, which implies that k_s must increase as well. We conclude that as k_y increases the graphs of f_b pile on top of each other and hence the graphs of φ_b lie below each other.

$k_{h1} = k_{h2} = 0$**:** First of all, an easy check shows that $(0, \overline{H}, 0, \overline{C}, 0, \overline{T}, 0, \overline{R}, 0)$ is a steady state for all values of k_s. But for k_s small enough, a second positive steady state exists as well.

In this case we have $\alpha = \overline{R}$ and

$$k_s = f_b(x_8) = \frac{k_6 x_9}{x_1}.$$

Hence both the numerator and denominator of f_b tend to zero as x_8 tend to $\alpha = \overline{R}$. By plugging the expression of x_1 into f_b, we have:

$$f_b(x_8) = \frac{k_6 k_{3r} k_{4r} k_y (\overline{C} - x_9) x_8}{k_3 k_4 (\overline{R} - x_8) - k_{3r} k_{4r} x_8}.$$

We deduce that when $x_8 = \overline{R}$, then $x_9 = 0$ and $f_b(x_8) = k_6 k_y \overline{C}$. It follows that $k_s = f_b(x_8)$ does not tend to infinity as x_8 approaches the upper bound of I, $\overline{R}$. In this case, the signal-response curve is defined by f_b^{-1} for $k_s \in [0, k_6 k_y \overline{C}]$ and is constant at $\overline{R}$ for $k_s > k_6 k_y \overline{C}$.

References

[1] E. Feliu, C. Wiuf, Simplifying Biochemical Models With Intermediate Species. To appear in Journal of the Royal Society Interface, 2013.

[2] H. Ji, P. Ellison, D. Knight, M. Feinberg, The chemical reaction network toolbox, version 2.2. http://www.chbmeng.ohio-state.edu/ feinberg/crntwin/, 2012.
